# Online Parent Training for The Initial Management of ADHD referrals (OPTIMA): the protocol for a randomised controlled trial of a digital parenting intervention implemented to support parents and children on a treatment waitlist

**DOI:** 10.1186/s13063-022-06952-z

**Published:** 2022-12-12

**Authors:** Katarzyna Kostyrka-Allchorne, Claire Ballard, Sarah Byford, Samuele Cortese, David Daley, Johnny Downs, Blandine French, Cristine Glazebrook, Kimberley Goldsmith, Charlotte L. Hall, Ellen Hedstrom, Hanna Kovshoff, Jana Kreppner, Nancy Lean, Kapil Sayal, James Shearer, Emily Simonoff, Margaret Thompson, Edmund J. S. Sonuga-Barke

**Affiliations:** 1 Department of Child and Adolescent Psychiatry, Institute of Psychiatry, Psychology & Neuroscience, King’s College London, 16 De Crespigny Park, London, SE5 8AF UK; 2 Department of Health Service and Population Research, Institute of Psychiatry, Psychology & Neuroscience, King’s College London, London, UK; 3 Centre for Innovation in Mental Health, School of Psychology, Faculty of Environmental and Life Sciences, University of Southampton, Southampton, UK; 4 Clinical and Experimental Sciences (CNS and Psychiatry), Faculty of Medicine, University of Southampton, Southampton, UK; 5 Solent NHS Trust, Southampton, UK; 6 Hassenfeld Children’s Hospital at NYU Langone, New York University Child Study Center, New York City, NY USA; 7 NTU Psychology, School of Social Science, Nottingham Trent University, Nottingham, UK; 8 Academic Unit of Mental Health & Clinical Neurosciences, Faculty of Medicine & Health Sciences, University of Nottingham, Nottingham, UK; 9 Centre for ADHD and Neurodevelopmental Disorders Across the Lifespan CANDAL Institute of Mental Health, University of Nottingham, Nottingham, UK; 10 Department of Biostatistics and Health Informatics, Institute of Psychiatry, Psychology & Neuroscience, King’s College London, London, UK; 11 Department of Child & Adolescent Psychiatry, Aarhus University, Aarhus, Denmark

**Keywords:** ADHD, Conduct problems, Oppositional defiant disorder, Waiting list, Digital intervention, Mobile app, Parent training

## Abstract

**Background:**

Children referred for attention-deficit/hyperactivity disorder (ADHD) often present with a broader pattern of conduct problems including oppositionality and defiance. This combination can be extremely stressful to parents, lower parents’ self-esteem and negatively impact family life. The National Institute for Health and Care Excellence (NICE) recommends that families receive support as soon as possible after their referral. However, as clinical services are overstretched, and traditional in-person parenting intervention programmes are expensive, families often must wait times a long time prior to receiving this vital input. To address this, we have created a digital parenting programme called STEPS. It is delivered as a mobile phone app providing a set of tools and resources that can be easily accessed at parents’ convenience. This study aims to evaluate the clinical and cost-effectiveness of STEPS in supporting parents of children with high levels of hyperactivity/impulsivity, inattention and conduct problems, who are waiting to be assessed by specialist children’s clinical services.

**Methods:**

*Online Parent Training for The Initial Management of ADHD referrals* (OPTIMA) is a two-arm superiority parallel randomised controlled trial with an internal pilot study. We aim to recruit 352 parents and their children, who have been accepted onto a waitlist in Child and Adolescent Mental Health Services or similar child health services. Parents who consent will be randomised 1:1 to either the STEPS or wait-as-usual (WAU) group. The trial will be conducted remotely (online and telephone) with measures taken at baseline and 3, 6, 9 and 12 months post-randomisation. The primary objective is to evaluate whether STEPS reduces the severity of children’s oppositional and defiant behaviour, as rated by parents, measured at 3 months post-randomisation compared to WAU.

**Discussion:**

Digital solutions, such as mobile phone apps, have potential for delivering psychological support for parents of children with clinical-level needs in a timely and inexpensive manner. This trial will provide data on the clinical and cost-effectiveness of the STEPS app, which could support the implementation of this scalable parenting intervention programme into standard clinical care and, ultimately, improve the outcomes for families of children referred to specialist child and adolescent health services.

**Trial registration:**

ISRCTN 16523503. Prospectively registered on 18 November 2021. https://www.isrctn.com/ISRCTN16523503

**Supplementary Information:**

The online version contains supplementary material available at 10.1186/s13063-022-06952-z.

## Administrative information

**Table:** 

Title {1}	Online Parent Training for The Initial Management of ADHD Referrals: A two-arm parallel randomised controlled trial of a digital parenting intervention implemented on a treatment waitlist.
Trial registration {2a and 2b}.	ISRCTN: 16523503
Protocol version {3}	01 July 2022; V.2.2
Funding {4}	National Institute for Health Research RP-PG-0618-20003. The funding body played no role in the study design and conduct.
Author details {5a}	Katarzyna Kostyrka-Allchorne; King's College London Claire Ballard; King's College London Sarah Byford; King's College London Samuele Cortese; University of Southampton David Daley; Nottingham Trent University Johnny Downs; King's College London Blandine French; University of Nottingham Cristine Glazebrook; University of Nottingham Kimberley Goldsmith; King's College London Charlotte L Hall; University of Nottingham Ellen Hedstrom; University of Southampton Hanna Kovshoff; University of Southampton Jana Kreppner; University of Southampton Nancy Lean; King's College London Kapil Sayal; University of Nottingham James Shearer; King's College London Emily Simonoff; King's College London Margaret Thompson; Solent NHS Trust Edmund J. S. Sonuga-Barke; King's College London (corresponding author)
Name and contact information for the trial sponsor {5b}	King’s College London Address: Room 5.31, James Clerk Maxwell Building, 57 Waterloo Road, London SE1 8WA Telephone: +44 (0)207 8483224 Email: reza.razavi@kcl.ac.uk The South London and Maudsley NHS Foundation Trust Address: R&D Office (POO5), Room W1.09, IoPPN Main Building, King's College London, De Crespigny Park, London SE5 8AF. Telephone: +44 (0)207 8480675 Email: slam-ioppn.research@kcl.ac.uk
Role of sponsor {5c}	The sponsors played no role in the study design.

## Introduction

### Background and rationale {6a}

Attention-deficit/hyperactivity disorder (ADHD) is a common neurodevelopmental condition characterised by symptoms of inattention and/or impulsivity-hyperactivity [1]. It is associated with a range of academic, employment and social negative outcomes and impairment [2–6]. In 2012, annual ADHD-related health, social care and education costs in the UK were estimated at £670 million [7]. These negative impacts are often driven by a broader pattern of common behaviour problems (e.g. oppositional, disruptive, defiant behaviours) in addition to the symptoms of inattention and hyperactivity/impulsivity themselves [8]. Effective multi-modal treatments for ADHD exist but access to them in the UK is currently limited by budgetary constraints and there are long waiting lists for specialist clinical assessment, diagnosis and treatment [9]. In 2014, a United Kingdom Parliament Health Committee reported that even maximum acceptable waiting times for complex cases of neurodevelopmental disorders such as ADHD were already as long as 15 weeks in 2013 [10]. In terms of actual waiting times, the Children’s Commissioner Lightning Review in 2016 found up to 200 days delay to initial assessment [9]. Prolonged waiting times may lead to families feeling unsupported during this stressful period and, importantly, to the escalation of difficulties, a decrease in motivation, poorer engagement and premature dropout from future treatment [11].

The goal of the *Online Parent Training for the Initial Management of ADHD referrals* (OPTIMA) is to provide support for families for 3 months during the period between referral acceptance and the first full clinical assessment. It focuses on supporting parents to better manage their children’s co-occurring behaviour problems. These present a major challenge to parents, increasing levels of their parenting stress [12] and therefore the likelihood of mental health problems [13]. They contribute to difficult or coercive child-parent relationships that may deteriorate over time [14]. It is the escalation of these behaviour problems to “crisis levels” that often lead parents with children who have ADHD-type problems to first seek specialist help [15]. For many parents finding a way to manage their child’s disruptive and defiant behaviour is likely to be the most urgent treatment priority at the time of their initial referral.

Effective ways to help parents manage these behaviour problems already exist—these come broadly under the term Parent Training (PT; (1)). Meta-analyses of randomised controlled trials (RCTs) show that PT improves parent-child relations and reduces challenging behaviour in children with ADHD, both as seen from the parents’ point of view and by independent observers who are blind to treatment assignment [16, 17]. PT is traditionally delivered face-to-face either in small groups or on a one-to-one basis, which means that it is costly to provide and time-consuming to organise [18]. PT is typically only offered after a full clinical assessment has been made and an ADHD diagnosis is given. This means that even in the best-case scenario parents can be left without support and guidance for long periods while waiting for the assessment process to take its course. This risks the further deterioration of the parent-child relationship and escalation of the child’s problems—with the concomitant probable effects on parent and family wellbeing that this implies. However, the National Institute for Health and Care Excellence (NICE) recommends that irrespective of the length of the waiting period before a full assessment is given, support should be made available to parents soon after their referral is made—allowing them to start addressing their children’s behavioural difficulties early on.

In OPTIMA, we address this challenge by employing a digital intervention, a parenting app, designed to provide parents on the waitlist with support and advice so that they can better manage their children’s behaviour. Three studies have examined digital PT for families with children showing ADHD-type problems [19–21]. Studies that compared online PT to a waitlist condition showed significant reductions in children’s problem behaviour [20] and an increase in parental competence, satisfaction in their parenting role and maternal wellbeing [21]. While these positive results are encouraging, small sample sizes in these studies and the different intervention content mean that their results should be interpreted with caution. Therefore, a large and adequately powered trial, such as OPTIMA, is required to provide further data on the effectiveness of digital parenting intervention programmes.

The present study will be an RCT to evaluate a new app called STEPS (Structured E-Parenting Support). Inspired by the New Forest Parenting Programme [22], STEPS was designed specifically to help parents manage the behaviour of their children referred with concerns regarding inattention and hyperactivity and impulsiveness (also described as ADHD-type), when used during the early post-referral/waitlist period. Key app objectives include the following: (i) increase parents’ knowledge of why their children behave in challenging ways, (ii) increase parents’ awareness of the importance of self-care and building their confidence as a parent, (iii) strengthen parent-child relationships and emphasise the importance of praise, (iv) facilitate effective communication patterns and (v) give parents practical needs-based guidance, strategies and text resources allowing the more effective management of their children’s behaviour. To keep costs to a minimum and to ensure that STEPS is a practical option that could be implemented within existing health service resources, there is no personal clinical support for parents using the app. However, information on how to access the appropriate clinical service if required (CAMHS or general practitioner) is provided within the Frequently Asked Questions accessible through the app Settings. Technical support is also provided for parents having difficulties with using the app. In OPTIMA, STEPS will be evaluated, against wait-as-usual (WAU), using a two-arm parallel randomised controlled superiority trial with an internal pilot. In line with the latest Medical Research Council guidelines for the evaluation of complex interventions [23], an embedded process evaluation will aim to explain the study outcomes and explore the contextual factors that might influence those outcomes. The process evaluation will be described in a separate protocol. The RCT will evaluate the clinical and cost-effectiveness of STEPS.

## Objectives {7}

The overall objective of the trial is to evaluate a digital app (STEPS) designed to help parents of children with high levels of hyperactivity/impulsivity, inattention and conduct problems, who are waiting to be assessed by Child and Adolescent Mental Health or other similar child health services.

### Primary objective

To evaluate whether children screened positive for high levels of hyperactivity/impulsivity, inattention and behaviour problems (oppositionality and defiance), STEPS reduces the severity of behaviour problems as rated by parents (primary outcome) measured at 3 months post-randomisation, compared to WAU.

### Secondary objectives

1. To test for the maintenance of STEPS’ effects on the primary outcome at 6, 9 and 12 months post-randomisation.
2. To test whether STEPS reduces the severity of parent-rated hyperactivity/impulsivity and inattention at 3- and 12-month follow-up, as compared to WAU.
3. To test whether STEPS improves parenting (i.e. style, satisfaction and efficacy), increases child-parent closeness and reduces parenting-related strain at 3- and 12-month follow-up, as compared to WAU.
4. To establish the cost-effectiveness of STEPS compared to WAU at 3 and 12 months post-randomisation with outcomes measured in terms of quality-adjusted life years.
5. To use qualitative data from parents and clinicians and quantitative data from parents and children to explore how STEPS works, what the mechanisms of change are, contextual factors influencing level of engagement with the app and potential moderators and mediators of change in ODD. This analysis will not form part of the main trial results. This mixed-methods approach will be applied in a way that is consistent with the Medical Research Council guidelines for complex intervention process evaluation research [23, 24].

### Exploratory objectives

1. To test whether STEPS reduces levels of child oppositionality and defiance expressed through speech (and rated by a blinded researcher) during a period of task-focused parent-child interaction at 3- and 12-month follow-up, as compared to WAU.
2. To examine whether STEPS reduces the severity of parent-rated emotional problems at 3- and 12-month follow-up, as compared to WAU.
3. To conduct an exploratory post-randomisation effect modifier analysis to examine whether app usage levels and patterns up to 3 months influence the effect of STEPS on the primary behaviour problems outcome at 3 months and the secondary behaviour problems outcome at 12 months.
4. To conduct an exploratory post-randomisation effect modifier analysis to see whether contact with clinical services up to 3 months post-randomisation influences the effect of STEPS on the primary behaviour problems outcome at 3 months and if clinical contact up to 12 months post-randomisation influences the effect of STEPS on the behaviour problems secondary outcome effect at 12-months.
5. To conduct an exploratory analysis to see if STEPS reduces the likelihood of a clinical diagnosis being made and medication prescribed by the 12-month follow-up.

## Trial design {8}

OPTIMA is a two-arm superiority parallel randomised controlled trial with an internal pilot study.

## Methods: participants, interventions and outcomes

### Study setting {9}

The trial will be conducted remotely (using phone calls and digital methods), with researchers based in three trial centres: London, Southampton and Nottingham. Across all trial centres, participants will access the intervention (the STEPS app) using their mobile device in their preferred setting.

### Eligibility criteria {10}

#### Inclusion criteria

- Parents (aged 18 years or older) of children aged 5 to 11 years, who have been accepted onto the assessment waiting list in selected services in England and their child referred for a mental health assessment. Given the differences in the referral pathways existing in the organisations supporting OPTIMA recruitment, all mental health and neurodevelopmental referrals will be initially accepted as eligible and screened. To be eligible, the child should have been on the waitlist for no longer than nine calendar months.
- The child scored ≥ 8/10 on the hyperactivity/inattention and ≥ 4/10 on the conduct problems subscale of the Strengths and Difficulties Questionnaire [25]. These cut-offs each identify the top 10% of the population [26].
- Self-perceived English language competence.
- The parent has access to a mobile phone using the iOS or Android operating system that connects to the internet.

#### Exclusion criteria

- A child living under local authority care.
- The child already received a clinical diagnosis of ADHD and/or received treatment for ADHD (pharmacological or non-pharmacological).
- Children with other pre-existing diagnoses will not be excluded.

If two children from the same family are referred during the trial at the same time and both meet inclusion criteria, then only the older of the two will be included. If they are referred at different times, the first child will be included.

### Who will take informed consent? {26a}

Participants in the study will be parents of children, who screened positive for high levels of hyperactivity/impulsivity, inattention and conduct problems; children themselves (parent-child interaction task only, optional); and clinicians (qualitative interviews only). The method for taking consent will be different for each of these three participant groups:

#### Parents

All parents will provide written informed consent to take part in the study. The informed consent form will be provided in electronic format using Red Pill ePRO system and will be completed by the participant before they enter the study. A link to the electronic consent form will be emailed or texted to parents who wish to enroll in the study. The electronic consent form will include a link to the *Parent Information Sheet*. On the consent form, there will be a separate question asking parents to consent to a qualitative interview. Parents, who agree to take part in the interviews via the written consent form, will be asked to confirm their consent verbally before the interview takes place.

#### Children

Consent statements regarding a child’s participation in the study will be included in the electronic consent form completed by parents via the Red Pill ePRO system. Parents, who consented to their children’s participation, will be emailed The Child Information Sheet. Children’s verbal assent will be obtained before each child-parent task session and will be documented in the trial database.

#### Clinicians

The informed consent form for clinicians will be provided in electronic format using Qualtrics. A link to the electronic consent form will be emailed to clinicians who wish to take part in the interviews. The electronic consent form will include a link to the Clinician Information Sheet.

### Additional consent provisions for collection and use of participant data and biological specimens {26b}

On the consent form, participants will be asked if they agree to allow the STEPS app to track its use and give permission for their usage records to be retrieved and used by the study team. Participants will also be asked for permission for the research team to share relevant data with researchers from the collaborating Universities. This trial does not involve collecting biological specimens for storage.

### Interventions

#### Explanation for the choice of comparators {6b}

No alternative evidence-based intervention is currently recommended, available, or implemented in the United Kingdom during the post-referral waiting period for patients waiting for a child health services assessment. WAU, therefore, is the most appropriate comparator. Those randomised to WAU will receive STEPS after they have completed the 12-month OPTIMA follow-up. In the sites across our three centres, given the current length of waiting lists in the UK, we do not expect any children to receive their clinical assessment and initiate treatment within the first 3 months of randomisation or few families, if any, to engage in self-initiated treatment during this period. Parents will not be stopped from initiating access to services over any of the 12 months of the trial.

#### Intervention description {11a}

**Fig. 1:**
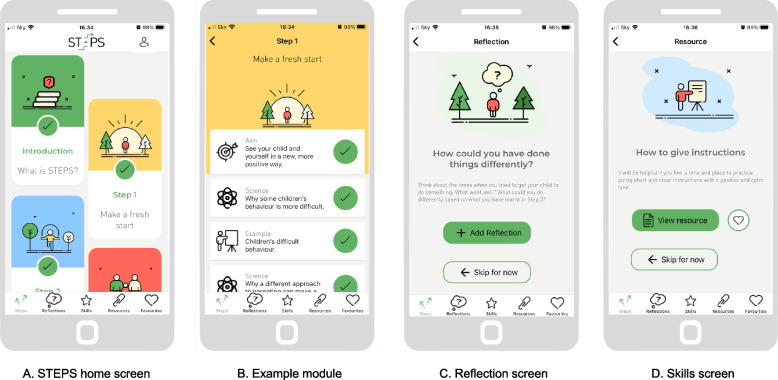
Screenshots of the STEPS app: **a** the app home screen; **b** example module screen; **c** reflection screen; **d** skills screen

STEPS (Structured E-Parenting Support; Fig. 1) is a mobile phone application (app) that provides a set of tools to support parents to manage their 5–11 years old children’s behavioural problems. Its content has been shaped by research about parenting and child behaviour and many years of clinical experience. The content is delivered using short videos and audio clips. There is no personal clinical support for parents using STEPS. The app also includes downloadable resources and space to record audio or written notes. The app aims to be flexible and convenient; parents can move through the eight modules (steps) at their own pace and in their own time. However, the order in which parents progress through the modules is fixed. It takes about 20 min to complete each module. For more detailed information about the content and structure of each module, see Kostyrka-Allchorne et al. [27]. STEPS works on most smartphones using the iOS or Android operating systems. It is available to download through the public app stores; however, access is controlled through the use of study IDs required to register on the app.

##### Frequency and duration of intervention

Participants in the treatment arm will have access to STEPS for 3 months after which they will no longer be able to use the app. STEPS is a self-guided intervention and the time needed to complete each module (step) will depend on the pace of the individual user. Completion of the two first modules: ‘Make a fresh start’ and ‘Look after yourself’ will constitute adherence to the intervention.

##### Intervention records

Parents’ use of the intervention will be recorded automatically within the app. Usage data will include the number of modules started; the number of completed modules—that is the number of modules where the parent progressed through all the mandatory elements contained within that module; the number of recorded reflections; the number of accessed resources; the number of items added to favourites; and the amount of time parents spent on watching videos or listening to audio skills.

#### Criteria for discontinuing or modifying allocated interventions {11b}

The risk of participants experiencing any adverse events during this trial because of using STEPS, and therefore the need to discontinue the intervention, is very low. If there is any indication that a participant has experienced harm due to taking part in the trial or the intervention, a decision of whether they should be withdrawn from the study will be taken by the Chief Investigator in consultation with the *Programme Steering Committee (PSC)*. Participants may withdraw from the trial or the intervention at any time.

#### Strategies to improve adherence to interventions {11c}

The app has an attractive design and is easy to use. Its usability has been tested with the *OPTIMA Patient and Public Involvement and Engagement Panel (PPIE)*. To promote engagement, automatically programmed digital reminders (text messages) will be sent from the app to participants reminding them to use STEPS. Levels of engagement will be monitored by evaluating the number of completed modules (steps)—data that are collected automatically by the app while it is being used.

#### Relevant concomitant care permitted or prohibited during the trial {11d}

There will be no restrictions on concomitant care, which will be monitored carefully during the trial through the service use questionnaire (CA-SUS [28];).

#### Provisions for post-trial care {30}

No post-trial care is planned for the study and participants will receive care as usual from the services they were referred to.

### Outcomes {12}

#### Child outcomes

##### Parent-rated behaviour problems (primary outcome at 3 months post-randomisation)

The primary outcome will be measured with the oppositional and defiant disorder (ODD) subscale of the *Swanson, Nolan, and Pelham Rating Scale–MTA version* (SNAP-IV [29];). The subscale consists of eight items that are rated on a 4-point scale (not at all, just a little, quite a bit, very much). The subscale score is obtained by averaging responses across the eight items. This SNAP-IV ODD subscale is a valid outcome measure for use in clinical trials [30].

Child disruptive and defiant behaviour was selected as the primary outcome because for many parents of children referred to clinical services, this is likely to be the most urgent treatment target at the time of their initial referral.

##### Parent-rated hyperactivity/impulsivity and inattention

This will be measured with the respective subscales of the SNAP-IV [29]. Each of these two subscales consists of 9 items that are rated on a 4-point scale (not at all, just a little, quite a bit, very much). The subscale scores are obtained by averaging across the 9 items associated with the subscale and can be further combined into a single ADHD scale score by deriving an average across the two subscales [30, 31]. The SNAP-IV ADHD scale has confirmed validity for use as an outcome measure in clinical trials [30].

##### Parent-rated emotional problems

This outcome will be measured by the relevant SDQ subscale [25]. This subscale measures fearfulness, anxiety and low mood and consists of 5 positively phrased items rated on a 3-point scale (not true, somewhat true and certainly true). Individual items’ scores are summed to derive an overall emotional problems subscale score.

NB: This outcome has been added via a substantial amendment after the start of recruitment, therefore, we have added it as an exploratory outcome. Baseline scores (T1) for all participants will be extracted from the medical records, using information from the SDQ completed nearest to the baseline assessment due date. Three- (T2) and 12-month (T5) data will be collected for most participants online via the Sealed Envelope platform. For any participants already enrolled in the trial and past the T2 timepoint by the time this measure is added, we will aim to extract T2 data from the medical records if a participant completed the SDQ as part of the routine clinical follow-up within the T2 visit window.

##### Independent observer-rated behaviour problems

Parents and children will be invited to jointly complete an online drawing task, Etch-a-Sketch Online. It is a newly developed and validated online tool that allows remote observation of parent-child interactions at home [32]. During the 5-min task, parents and children will take turns drawing a simple picture on a mobile phone screen. The task will be audio recorded and the parent-child verbal exchange will be rated using the *Child Oppositional and Defiance Speech Sample* (CODSS), which has been developed specifically to be used in the present study. CODSS captures the level of four problematic aspects of child behaviour (i.e. being argumentative, defiant, easily annoyed, angry) by an independent researcher using a 5-point scale (not at all, a little, moderately, very, extremely). The overall rating score will be derived by averaging across the items.

#### Parent and family outcomes

##### Parenting style: laxness and over-reactivity

Laxness captures a lack of consistent responding; over-reactivity captures overly emotional or harsh responses [33]. These outcomes will be measured with the respective 5-item subscales from The O’Leary *Parenting Scale* [34]. The probability of using specific parenting strategies in response to child misbehaviour is rated on a 7-point scale and is anchored by one effective and one ineffective response strategy. Responses to individual items are summed up to derive an overall subscale score.

##### Parenting satisfaction and efficacy

Parenting satisfaction will be measured with a 9-item subscale from the *Parental Sense of Competence Scale* (PSCS [35];), which has good validity [36]. Parenting efficacy reflects parents’ perceived competence, capability and problem-solving abilities as a parent and will be measured with a 7-item subscale from the PSCS [35]. The subscale has established validity [37].

The respective subscale items are positively framed (efficacy) or negatively framed (satisfaction), and parents make responses on a 6-point scale, with options ranging from “strongly disagree” to “strongly agree”. The respective subscale scores are calculated for each participant by summing up individual item scores.

##### Parenting-related strain

This will be measured with the global score obtained on the *Caregiver Strain Questionnaire* (CGSQ [38];). CGSQ consists of 21 items with responses made on a 5-point scale ranging from “not at all” to “very much”. Three indices are calculated for each participant by averaging individual item scores for the three subscales: Objective Strain (11 items), Subjective Internalised Strain (6 items) and Subjective Externalised Strain (4 items). Global Caregiver Strain Score is determined by calculating the sum of the three subscale scores. The scale is a reliable and valid measure of parenting-related strain (38).

##### The closeness of the child-parent relationship

This will be measured with the closeness subscale of the *Child-Parent Relationship Scale – Short Form* (CPRS [39];). This 7-item subscale scale measures the extent to which parents feel that their relationship with a child is characterised by warmth, affection and open communication. It has good validity for measuring child-parent closeness [40]. Responses are made on a 5-point scale ranging from “definitely does not apply” to “definitely applies”. The items are summed to obtain a single subscale score.

#### Other measures

##### Characterisation of the child’s behaviour problems

This will be measured with the *Child’s Challenging Behaviour Scale version 2* (CCBS [41];). The CCBS is a 9-item measure of challenging behaviours for children aged 5–18 years. Parents rate agreement with statements about their child’s behaviour on a 4-point scale ranging from “strongly agree” to “strongly disagree”, and the total score on the CCBS is calculated by summing the scores.

##### Autism spectrum disorder (ASD) symptoms

This will be measured with the *Social Communication Questionnaire*—lifetime version [42]. The SCQ is a 40-item screening measure for autism spectrum disorder and is validated for use with children ages 4 years and older. Questions focus on behaviours that are likely to be observed by the primary caregiver and concern the following domains: reciprocal social interactions, language and communication and repetitive and stereotyped patterns of behaviours. The presence of autism behaviours is coded as 1 and its absence as 0. The first question concerns the level of current language and is not included in the total score. For children with language, all questions apply, and the maximum score is 39; for children without language, the first 6 questions do not apply so the maximum score is 33 (language items are inapplicable).

##### Family characteristics and demographic measures

Parents will provide information about their child’s age and sex, their own sex, their own and their child’s ethnicity, parental education and employment and relationship status and whether there are other children with neurodevelopmental difficulties living in the household. We will estimate family socioeconomic status based on total household income, coded as < £16,000 | £16,000–£29,999 | £30,000–£59,999 | > £60,000 | Prefer not to say.

##### App usage data

To establish intervention adherence, the number of completed modules will be measured (min = 0; max = 8), with the completion of the first two modules constituting adherence to the intervention. Other collected app usage events will include the number of started modules, the number of videos watched, the time spent watching videos (in seconds), the number of audio clips listened to and the time spent listening to audio clips (in seconds), the number of reflections recorded, the number of items saved to favourites and the number of accessed text resources. These will be used to provide descriptive information about app usage patterns. The format of the app usage data is being finalised as part of the further app development, so we are not yet able to specify the app usage variable(s) up to T2 that will be used as the effect modifier. Likely options include the number of modules started or the total time engaging with the app. We will aim to specify this in the statistical analysis plan.

##### Clinical diagnosis

Information about ADHD diagnosis and any pharmacological treatment for neurodevelopmental and mental health disorders will be extracted from medical records at 12-month follow-up. In case it is not possible to access a child’s medical record, information will be collected directly from a parent (via a phone call). The presence of an ADHD diagnosis will be coded as 1 and absence as 0. In addition, if another diagnosis has been given, information about the type of diagnosis will be recorded for descriptive purposes. Similarly, pharmacological treatment for mental health disorders will be coded as 1 and its absence as 0. If pharmacological treatment has been prescribed, information about the name of the drug and dose and a reason for prescribing it will be recorded for descriptive purposes.

##### Trial expectations

Information about the participants’ expectations about parent training in general as well as the specific expectations about the STEPS app will be collected using a questionnaire developed specifically for the study (see Additional file 1). Parents will be asked to rate the statements on the Likert-type scale as well as to provide open-ended text responses.

##### Experience of parenting

This will be measured with open-ended questions: “Think of a memorable interaction that you have had with your child within the last 24 hours. Tell us about that interaction. For example, what went well and why or what went badly and why? What might you do differently next time?”

##### Adverse events

This will be measured with the *Medical and Psychological Events and Difficulties Questionnaire* (MAPED), which has been developed for the study (see Additional file 1). Parents will be asked to report retrospectively any physical and/or mental health difficulties they or their child have experienced in the last three months. They will also be asked to report any difficulties they or their child have had with daily activities. These will be used to monitor safety and to provide descriptive information about adverse events experienced during the trial.

#### Economic measures

##### Resource-use data

This will be measured using the *Child and Adolescent Service Use Schedule*, a measure that has been applied in a range of populations of young people with mental health problems [43, 44] The CA-SUS collects information on the use of all hospital and community-based health and social care services, including health and social care services provided within education settings, service-provided accommodation (for example, Local Authority foster or residential care) and prescribed medications for mental health conditions.

##### Child’s health-related quality of life

This will be measured with the *Child Health Utility* measure [45]. The CHU9D is a paediatric preference-based quality of life measure for use in healthcare resource allocation decision-making. The CHU9D has been designed for self-report by children aged 7 to 17, but with an interviewer’s help, can also be used in children as young as 6 years old [46] and guidance is available from the developers for proxy completion by parents for children aged 5 and under. The current study, however, does not involve collecting questionnaire data from children and thus the CHU9D will be proxy completed by the parents/carers of all young participants using the proxy version of the measure. The questionnaire includes 9 items, each with a 5-level response category. Each item focuses on a different domain of children’s present functioning: worry, sadness, pain, tiredness, annoyance, school, sleep, daily routine and activities.

##### Parental health-related quality of life

This will be measured with the *EQ-5D-5L* [47]. This questionnaire captures general health across five domains: mobility, self-care, usual activities, pain/discomfort and anxiety/depression. Each domain has five levels (no problem, slight problem, moderate problem, extreme problem and severe problem/unable to complete activities).

### Participant timeline {13}

**Fig. 2:**
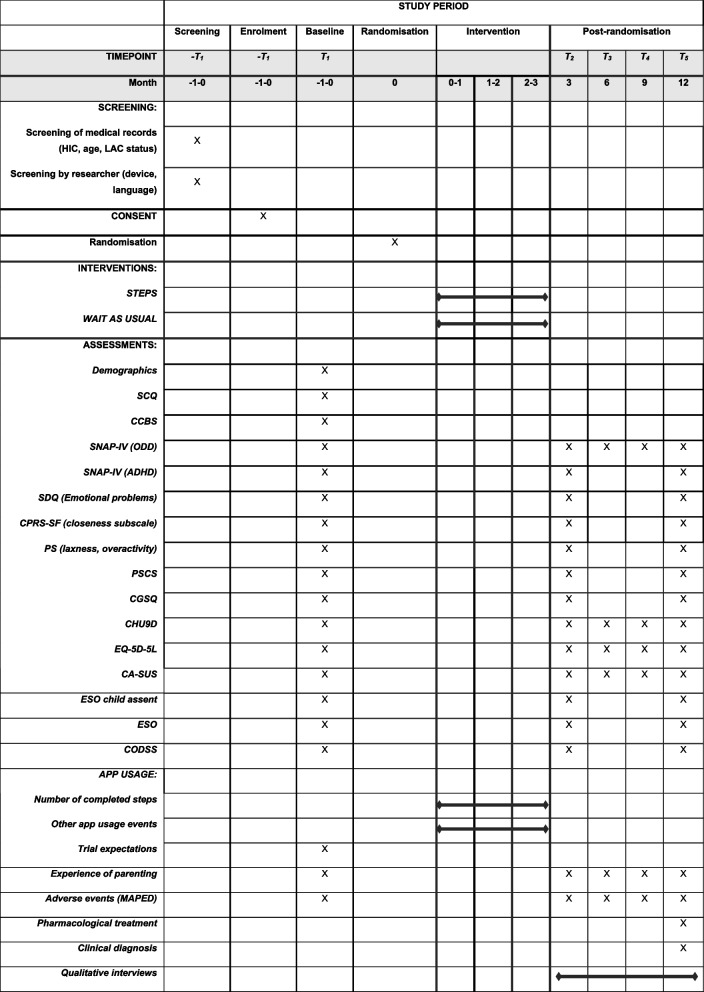
OPTIMA RCT schedule of assessments. HIC, high levels of hyperactivity/impulsivity, inattention and conduct problems; LAC, child in local authority care; SCQ, The Social Communication Questionnaire; CCBS, Child’s Challenging Behaviour Scale version 2; SNAP-IV, The Swanson, Nolan, and Pelham Rating Scale – the MTA version; ODD, oppositional defiant disorder; ADHD, attention-deficit/hyperactivity disorder; SDQ, The Strengths and Difficulties Questionnaire; CPRS-SF, The Child-Parent Relationship Scale-Short Form; PS, The O’Leary Parenting Scale; PSCS, The Parental Sense of Competence Scale; CGSQ, The Caregiver Strain Questionnaire; CHU9D, Child Health Utility; CA-SUS, The Child and Adolescent Service Use Schedule; MAPED, Medical and psychological events and difficulties questionnaire; CODSS, Child Oppositional and Defiance Speech Sample Scale; ESO, Etch-a-Sketch Online

Measures will be taken at baseline (T1), scheduled within 1 month before randomisation and then at 3 months (T2 primary outcome, primary timepoint), 6 months (T3), 9 months (T4) and 12 months (T5) post-randomisation. Figure 2 details screening and assessment details at each timepoint.

### Sample size {14}

A total of 352 parents and their children will be recruited into the study. This power calculation is based on projected effects at the primary endpoint (3 months post-randomisation). As behaviour problems measured with the ODD subscale of the SNAP-IV questionnaire [29] is our primary outcome, we estimated the smallest difference of clinical importance between STEPS and WAU to be equivalent to an effect size d = 0.4 standard deviations based on the NICE guidance supporting the use of PT for the treatment of ODD or conduct disorder [48]. A within-trial drop-out rate of 25% is assumed. This is higher than in most previous trials of face-to-face PT programmes because of the unsupported nature of STEPS in OPTIMA. We will monitor drop-out rates during the internal pilot and, if necessary, recalculate the sample size if it is higher than the 25% estimated. Using Stata (version 14.0) command sampsi, ANCOVA analysis with a conservative zero correlation assumed between baseline and primary endpoint SNAP-IV ODD score, a two-sided test and an alpha of 0.05, we estimated that 176 individuals will be needed per trial arm (total *n* = 352) to provide 90% power to test the hypothesis that STEPS is superior to WAU.

### Recruitment {15}

Initially, recruitment of parents will take place across five sites delivering secondary (community paediatrics) and secondary/tertiary (child and adolescent mental health and behavioural support services) care and support for children’s behavioural and mental health problems. These sites are in urban areas with catchment populations from a range of socioeconomic backgrounds. However, more sites will likely join during the trial. A complete up-to-date list of study sites will be available on the OPTIMA website www.optimastudy.co.uk and will be reported in the main trial publication.

Identification of potentially eligible parents will occur mainly via myHealthE, a Caldicott Guardian approved, General Data Protection Regulation (GDPR) compliant online portal for the automated screening of referred families using NHS CAMHS data [49, 50]. Using this method will allow quick and efficient screening and obtaining of consent-for-research-contact from families on the waitlist that have very limited contact with the clinicians, who usually act as gatekeepers to referring families to research studies. Other digital (i.e. Interactive CAMHS Assessment Network, ICAN) and non-digital methods of identification (i.e. manual clinical records review) will also be used. All these approaches have been described in detail in the previous publication [27].

Clinicians taking part in the qualitative interviews (*n* ~ 10) will be recruited from the services involved in the study. Service managers will be approached with a request to circulate the Clinician Information Sheet to the members of the team and the clinicians interested in taking part will be asked to contact the research team directly.

## Assignment of interventions: allocation

### Sequence generation {16a}

Once the parent has consented and completed the baseline assessments, they will be randomised to either the STEPS or WAU. Randomisation will be carried out online via a randomisation platform provided by *Sealed Envelope* in a 1:1 ratio and stratified by trial centre location (London, Nottingham, Southampton) using random permuted blocks procedure with varying block sizes.

### Concealment mechanism {16b}

To ensure allocation concealment, a centralised service provided by *Sealed Envelope* will be used. It will not be possible for researchers responsible for randomisation to know the allocation sequence in advance.

### Implementation {16c}

The designated trial administrators will access the randomisation service and complete an electronic form and the randomisation allocation will be released. The parent will be notified of the treatment allocation via email or by phone by the researcher who performed randomisation.

## Assignment of interventions: blinding

### Who will be blinded {17a}

Parents taking part in the study will remain unblinded throughout the trial; they will be informed about their group allocation after randomisation. Research assistants will remain blinded throughout the trial. The senior statistician and senior health economist will remain fully blinded until a review of the first draft of the final statistical/health economic reports for checking when they will become fully unblinded. Similarly, the Chief Investigator and Principal Investigators in each trial centre will remain fully blinded until they review the finalised statistical report when they will become fully unblinded. The junior statistician and junior health economist will be partially blinded until sign-off of the statistical and health economic analysis plans, after which they will be fully unblinded so they can inspect and utilise app usage/therapy-related data. The trial manager and the trial administrators will be unblinded. The only individuals that will be able to summarise/see data by arm before the review of the statistical report are the junior statistician, junior health economist and the members of the data monitoring committee.

### Procedure for unblinding if needed {17b}

No serious harms associated with taking part in the intervention are expected; therefore, a formal procedure for unblinding any blinded staff during the study is not needed.

## Data collection and management

### Plans for assessment and collection of outcomes {18a}

All OPTIMA trial data will be collected remotely: online, by phone and via video/audio chat. For each participant, assessments will take place over 12 months and will be conducted at five timepoints as described above [baseline (T1), T2, T3, T4, T5].

Four categories of data will be collected in the study:

1. Participants’ reports of the outcome measures and information about adverse events, which will be collected online using Red Pill—a secure electronic data capture and management system provided by Sealed Envelope—or over the phone by a trained researcher. Red Pill will also be used to record data entered directly by researchers, for example, the CODSS ratings or information about diagnosis and treatment.
2. A speech sample will be audio-recorded during a remote parent-child drawing task (ESO) conducted via Microsoft Teams.
3. The STEPS app usage data will be collected within Google Firebase and will be stored with Google servers.
4. Video and audio recordings of qualitative interviews. Whenever possible, remote interviews will be conducted and recorded via Microsoft Teams. In case this is not possible, participants will be given an option to take part in a telephone interview, which will also be recorded with Microsoft Teams. Alternatively, a researcher will email a participant a list of questions, and they will be asked to respond in text form.

### Plans to promote participant retention and complete follow-up {18b}

In the first instance, participants will be notified by email and/or a text message when a scheduled assessment requires them to complete the questionnaires online using the participant-entered forms (ePRO) system provided by *Red Pill*. They will be able to complete the questionnaires using the browser on their computer or phone. Researchers will be able to track questionnaire completion within *Red Pill* using a display listing both complete and incomplete questionnaires for each participant. Up to three emails/text messages will be sent as reminders to complete online forms. Non-responders will be followed up with phone calls throughout the assessment window. Participants will be informed that, if they wish, they can also complete the questionnaires or provide information about adverse events over the phone.

As a thank you for taking part, parents will receive shopping vouchers: £10 at baseline, £25 voucher at 3 months (T2) and £10 voucher after completing 6- (T3), 9- (T4) and 12-month (T5) assessments. Children will receive a £5 voucher and a certificate for each completed task.

### Data management {19}

The main trial database for the study will be provided by *Sealed Envelope*, which will ensure there are robust processes for quality management, security and backup of data https://www.sealedenvelope.com/security/. Access to the database and electronic data capture forms will be restricted by user identifiers and passwords to a limited number of researchers (i.e. trial manager and trial administrator, junior statistician, research assistants). Blinded researchers will not be able to view randomisation information within the database and will not have access to the forms that may provide unblinding information (e.g. experience of parenting or MAPED).

*Sealed Envelope* uses *Red Pill*, an online application for collecting and managing case report form (CRF) data on participants recruited to a clinical trial or other research studies. In this study, *Red Pill* will be used to record data collected offline (e.g. phone or medical records) as well as collect data directly from parents themselves [electronic patient-reported outcomes (ePRO)]. The system used in the OPTIMA trial will be specifically configured for the study.

Handling of all data collected in the OPTIMA trial is described in detail in the *OPTIMA Data Management Plan* (available from the study team). In brief, during the study, extracts of research data and the app usage data will be periodically downloaded from the respective databases (i.e. *Red Pill* and *Google Firebase*). These data will be de-identified and will be stored separately from any identifying information on King’s College London cloud storage infrastructure (*OneDrive for Business* or *Microsoft SharePoint*). The data stored on KCL cloud storage infrastructure are encrypted and access to the data will be restricted only to those who need to have it. All data will be handled in line with the institutional information governance policies and will follow the GDPR guidelines.

### Confidentiality {27}

The Chief Investigator and all members of the research team will take every effort to preserve the confidentiality of participants taking part in the study. To de-identify the data, each participant will be assigned a study ID. Participants’ identifiable data required for administrative purposes (e.g. name and contact details) will be stored in a separate file from the data files. These will be accessed only by those members of the research team who are responsible for contacting participants (e.g. to email a link to the online survey) and will be password protected. No individual participant’s data will be identifiable in the publications or reports that may result from this study.

### Plans for collection, laboratory evaluation and storage of biological specimens for genetic or molecular analysis in this trial/future use {33}

There will be no biological specimens collected in this trial (also see 26b).

## Statistical methods

### Statistical methods for primary and secondary outcomes {20a}

The main analysis will follow the intention to treat (ITT) principle [51, 52]. A mixed-effects linear analysis of covariance (ANCOVA) model with repeated measures will be used with the SNAP-IV ODD scores at 3 (primary outcome), 6, 9 and 12 months (secondary outcomes) post-randomisation as the dependent variables, and intervention group, time point, intervention group by time point interaction, baseline SNAP-IV ODD score, and the trial centre stratification variable as independent variables, with the interaction term used to extract the primary and secondary outcome STEPS vs WAU mean differences and associated 95% confidence intervals at the four timepoints. Similar statistical models will be used to test for intervention effects on the other continuous secondary (parent-rated hyperactivity/impulsivity and inattention, parenting style, satisfaction and efficacy, child-parent closeness and parenting-related strain) and exploratory (average levels of directly observer-rated child behaviour problems (CODSS), parent-rated emotional problems) outcome scale scores. For the binary exploratory diagnosis and medication outcomes, we will use modified Poisson regression with robust standard errors to estimate STEPS vs WAU relative risks (and associated 95% confidence intervals) for getting a diagnosis and being prescribed medication [53].

In addition to ITT analysis, we will undertake a complier average causal effect (CACE) sensitivity analysis [54, 55] on the ODD primary outcome at 3 months (T2) and ODD secondary outcome at 12 months (T5), to estimate treatment effects in those who completed at least two modules of the STEPS app (i.e. those that complied with the intervention).

Further detail of the analyses described in this section will be provided in the statistical analysis plan.

### Interim analyses {21b}

The study does not have a formal interim analysis planned but will include an internal pilot study (the first 9 months of recruitment). The objective of the internal pilot is to determine whether recruitment, engagement with the intervention and retention to the trial are sufficient to allow the trial to progress and provide a definitive answer on the effectiveness of the intervention. Progression rules regarding recruitment, treatment engagement and attrition during the internal pilot are specified in Table 1. Briefly, green means that the trial will continue; amber—the research team will review ways of improving with the PSC and the *OPTIMA PPIE* panel; and red—the trial will be stopped unless there are exceptional mitigating circumstances. The decision to continue or stop the trial will be made independently by the PSC taking advice from the Data Monitoring Committee.

**Table 1:** OPTIMA RCT internal pilot progression rules

**Recruitment**	
** Green: > 70%** of recruitment target for that period. ** Amber: 50–70%.** A lower rate would be also acceptable, if there is evidence of an upward recruitment trajectory and/or a clear plan for further improvement (e.g. new sites, or ways of recruiting). ** Red: < 50%** and no evidence of an upward trajectory.	
**Intervention engagement**	
** Green: > 90%** of participants providing primary outcome data at primary endpoint (3 months post-randomisation) will have engaged with the two first modules of the intervention. ** Amber**: **60–90%.** A lower rate also acceptable if there is evidence of improvement or/and a clear plan for improving engagement. ** Red**: **< 60%** and no evidence of improvement.	
**Attrition**	
** Green: > 75%** of participants who have reached the relevant time window will have completed primary endpoint assessments to provide primary outcome data. ** Amber: 50–75%.** A lower rate is acceptable if there is evidence of improving retention and/or a clear plan for enhancing retention. ** Red: <50%** and no evidence of improving retention.	

### Methods for additional analyses {20b}

#### Exploratory post-randomisation effect modifier analysis

Two exploratory post-randomisation effect modification analyses will be conducted. First, we will analyse the impact of the app usage (likely in terms of a number of completed modules or total time spent in the app) collected up to T2 on the effects of STEPS on the primary ODD outcome measured at 3 months post-randomisation (T2) and the secondary ODD outcome measured at 12 months (T5). Second, the impact of clinical contact/care: up to 3 months (T2) on the effects of the STEPS app on the ODD primary outcome at 3 months post-randomisation, and up to 12 months post-randomisation (T5) on the effects of the STEPS app on the ODD secondary outcome at 12 months. Clinical contact/care will include any treatment during the trial up to the two specified time points. The clinical care variables used as post-randomisation effect modifier will be extracted from the service use form (the CA-SUS) and will likely be somewhat post hoc as we are not currently sure which variables are most appropriate; this approach will be made clear to the reader in the subsequent publication. We plan to use appropriate methods for post-randomisation variables to explore whether the effects of interventions differ by these variables, such as principal stratification analysis [55]. Further details will be provided in the statistical analysis plan.

#### Health economic analysis

The economic evaluation will adopt the *National Health Service* (NHS)/personal social services perspective preferred by NICE, including health and social care services provided in education settings services, given the age of the population. Resource-use data collected using the CA-SUS will be costed using nationally applicable unit costs (e.g. P*ersonal Social Services Research Unit Costs of Health and Social Care*, *NHS Reference Costs, British National Formulary* for medications). The STEPS app will be costed in consultation with the application developers. Patterns and potential mechanisms influencing missing data will be explored to inform appropriate methods for handling missing data such as multiple imputation.

The primary economic evaluation will be a cost-utility analysis carried out at 3 months post-randomisation with outcomes expressed in terms of quality-adjusted life years (QALYs), using the proxy version of the CHU9D completed by a parent. Secondary economic analyses will include (i) a cost-utility analysis at 12 months post-randomisation to assess cost-effectiveness after formal assessment and treatment, (ii) a cost-effectiveness analysis undertaken at both 3 months and 12 months using the primary clinical measure of outcome (SNAP-IV ODD score) and (iii) a cost-utility analysis undertaken at both 3 months and 12 months combining QALYs for both the young person (using the CHU9D) and the primary parent/caregiver (using the EQ-5D-5L). Appropriate sensitivity analyses will be carried out, dependent on any assumptions made about the costing of the intervention, the method of measurement of outcomes or the approach to combining QALYs for young people and their primary carer.

Costs and outcomes will be compared at the 3-month and 12-month follow-up points and presented as mean values by the trial arm with standard deviations. Mean differences in costs and 95% confidence intervals will be obtained by non-parametric bootstrap regressions to account for the non-normal distribution commonly found in economic data. To provide more relevant treatment-effect estimates, analyses will include an adjustment for baseline covariates [56], which will be pre-specified and in line with the clinical analyses. Cost-effectiveness will be assessed using standard net-benefit approaches [57]. A joint distribution of incremental mean costs and effects for the two groups will be generated using non-parametric bootstrapping to explore the probability that each of the treatments is the optimal choice, subject to a range of possible maximum values (ceiling ratio) that a decision-maker might be willing to pay for an additional QALY (or unit improvement in behaviour problems). Cost-effectiveness acceptability curves will be presented by plotting these probabilities for a range of possible values of the ceiling ratio [58]. These curves are a recommended decision-making approach for dealing with the uncertainty that exists around the estimates of expected costs and expected effects associated with the interventions under investigation and uncertainty regarding the maximum cost-effectiveness ratio that a decision-maker would consider acceptable.

#### Process evaluation

The process evaluation will follow the *Medical Research Council* guidelines for evaluating the implementation of complex interventions [23, 24] and will be described in more detail in a separate protocol. Briefly, the process evaluation will use baseline data and post-intervention quantitative trial data (e.g. app usage data, including counts and time), qualitative data from semi-structured interviews with parents and clinicians (e.g. expectations, perceptions of impact, barriers to engagement) and textbox responses to open questions to explore the mechanisms for intervention engagement and impact (child behaviour change).

All parents will be invited to respond to questions about (1) their expectations of the trial and (2) accounts of their experience of parenting during trial participation. Post-intervention, researchers will conduct remote (telephone, video call or email) in-depth, semi-structured interviews with a subgroup of parents in the intervention group (*n* ~ 50). Maximum variation, purposive sampling will be used to ensure that a full range of views and experiences are captured (taking account of demographic factors and levels of app engagement). In addition, telephone interviews with clinicians (*n* ~ 10) will explore perceptions of the app and its perceived impact on preparing families for the formal clinical assessment. The interview schedules will be developed with Patient and Public Involvement (PPI) panel input. Interview data will be audio-recorded and analysed using thematic analysis [59], applying a framework approach [60].

### Methods in analysis to handle protocol non-adherence and any statistical methods to handle missing data {20c}

Missing data will be dealt with by using maximum likelihood methods to fit the mixed models described in the “Statistical methods for primary and secondary outcomes {20a}” section, with baseline variables predicting missing data included in the models. We will consider performing multiple imputation (MI) for primary and secondary outcomes only if there are post-randomisation variables that are predictive of missingness for these measures, in particular the measure of adherence to the intervention described in the “Intervention description {11a}” section, and the proportion of participants with missing values for any of the outcome variables is equal to or greater than 10%.

### Plans to give access to the full protocol, participant-level data and statistical code {31c}

The full protocol is available in Additional file 1. Requests for data and statistical code should be directed to the corresponding author.

## Oversight and monitoring

### Composition of the coordinating centre and trial steering committee {5d}

The Programme Steering Committee (PSC), a body independent of the research team, chaired by Professor Tamsin Ford, will provide formal oversight and expert advice for the overall OPTIMA programme, which includes oversight over the present trial. Its role is to ensure that the trial is conducted in a rigorous and timely manner and consider any proposed changes to the agreed programme of research. The PSC consists of an independent chair, statistician, digital health expert, digital mental health interventions expert, two parents of a child with ADHD and a health economics expert.

### Composition of the data monitoring committee, its role and reporting structure {21a}

The Data Monitoring Committee (DMC) is an independent body of experts, chaired by Professor Chris Metcalfe, that has been established to monitor the quality of trial data and the safety of participants. The DMC will have access to unblinded data if they wish. The DMC will be responsible for monitoring the overall conduct of the study, including recruitment, protocol compliance, accuracy and completeness of data collection. Based on this information, the DMC will make recommendations to the PSC, the Funder and the Sponsor on whether the study should continue or whether there are any ethical or safety reasons why the study should be modified or terminated. Any key changes to the study design and methodology will be reviewed by the DMC. The members of the DMC are completely independent of the trial and consist of an independent chair, statistician and clinical expert. The DMC will agree to a DAMOCLES charter, and the PSC will agree to terms of reference to outline their tasks and responsibilities.

### Adverse event reporting and harms {22}

The risk of participants experiencing any adverse events during this trial as a result of using STEPS is very low. Adverse events concerning parental or child physical and mental health will be monitored throughout the trial. Any physical or mental health difficulties spontaneously disclosed by a parent in their communication with researchers will be entered into the *Red Pill* database. Moreover, at each timepoint, participants will be asked to complete a formal questionnaire on adverse events (MAPED) that happened to them and/or their child. This information will be collected online using ePRO. The forms will be reviewed regularly, and all adverse events will be recorded on the OPTIMA adverse events form in the *Red Pill* database. All participants that experience a serious adverse event will be followed-up by the researchers, who have completed Level 2 safeguarding training, until the event is resolved. Where necessary, the participant’s clinical service which accepted the referral will be informed about the event.

### Frequency and plans for auditing trial conduct {23}

The Project Management Group will meet monthly to review trial conduct. The PSC will meet approximately every 6 months, with one meeting coinciding with the end of the internal pilot to provide advice to the funder whether the trial should continue. The DMC will also meet approximately every 6 months, and each meeting will be scheduled shortly before a PSC meeting.

### Plans for communicating important protocol amendments to relevant parties (e.g. trial participants, ethical committees) {25}

In case of new information becoming available, which may result in significant changes to the risks and benefits of taking part, the *Participant Information Sheet* and informed consent form will be reviewed and updated accordingly. All participants actively enrolled in the study will be informed of the updated information and will be given a revised copy of *the Participant Information Sheet* and informed consent form to confirm their wish to continue taking part.

Any changes to the protocol will be clearly communicated to the sponsor and the research ethics committee that approved the study as well as to the participating sites. A copy of the revised protocol will be sent to the local Principal Investigator to add to the Investigator Site File. Any deviations from the protocol will be fully documented in the Trial Master File.

## Dissemination plans {31a}

A complete account of the trial results will be published in a high impact peer-reviewed scientific journal and a full report for the funder. Authorship will be determined according to COPE standards based on the individual contributions. There will be no dissemination of the primary trial results until they are accepted for publication in peer-reviewed journal. In addition, the findings will be disseminated through oral and poster presentations at a range of conferences and seminars in the UK and overseas. There will also be a general dissemination programme for clinicians, commissionaires and parents through the OPTIMA website.

## Discussion

There is substantial evidence supporting the use of parenting intervention programmes in relation to children up to the age of 11 years, who present to clinical services with behaviour difficulties [61, 62]. Moreover, group and individual parent training sessions are included in the NICE clinical guidelines for the management of behavioural disorders in children and young people [63]. However, parents face substantial difficulties in accessing this kind of help, with a long waiting list being one of the key barriers. A mobile phone parent training app, such as STEPS, has the potential to provide a low-cost solution to enable parents to have timely access to psychological interventions and, potentially, to reduce the risk of problems escalating before an in-person clinical appointment becomes available. More generally, mobile phone affordances, such as its availability in almost any place and at any time and the privacy of users, could also help to overcome other barriers to access, for example, the lack of time or resources to attend an in-person session or stigma. Finally, the restrictions on face-to-face intervention delivery introduced at the time of the Covid-19 pandemic have inadvertently accelerated the adoption of digital solutions across many domains, including health care. Yet, there remains a substantial, but unrealised, potential in terms of using digital technology to provide psychological support for parents and children because of the lack of relevant evidence.

In the OPTIMA trial, we will test the clinical and cost-effectiveness of a mobile phone app called STEPS that provides a new, digital way to deliver parent training to families on children’s services waitlist. We will also examine the app usage data and qualitative information from semi-structured interviews with parents and clinicians to explore the mechanisms for intervention implementation and impact. Findings from this trial have the potential to add to the existing evidence base regarding applications of parent training and to help improve service delivery for families. The inclusion of several sites and different ways of identifying the potentially eligible families (i.e. digital vs traditional methods) will allow us to generalise our findings to a range of organisations and services. While this trial will provide data on the efficacy of STEPS in the group with clinical-level needs, future research should examine whether the app could prove effective in helping other populations, for example, families who do not meet the threshold for acceptance by specialist services.

In summary, the OPTIMA trial has the potential to inform whether parent training can be delivered effectively in a digital format and without personal clinical support. This study is also applying new digital methods to identify potentially eligible families quickly and efficiently and, thus, can serve as an example for future research.

## Trial status

Protocol version number V.2.2 dated 01 July 2022, recruitment started in May 2022 and is anticipated to be completed in December 2023.

## Supplementary Information

**Additional file 1.** Full study protocol.

## Data Availability

Anonymised data collected in the study will be suitable for sharing. Participants will be informed about plans for data sharing in the study information sheet and will agree to this when consenting to participate in the trial. To ensure all data can be shared, only participants who consent to data sharing will be eligible to participate in the trial. A decision of whether anonymised research data will be made publicly accessible or whether access will be controlled via data sharing agreements will be determined before depositing data in the suitable long-term storage repository and will be in line with the funder’s policies on data sharing procedures at that time.

## Abbreviations

ADHD: Attention-deficit/hyperactivity disorder
ANCOVA: Analysis of covariance
ASD: Autism spectrum disorder
CAMHS: Child & Adolescent Mental Health Services
CA-SUS: Child & Adolescent Service Use Schedule
CHU9D: Child Health Utility measure
CI: Chief Investigator
DMC: Data Monitoring Committee
EQ-5D-5L: EuroQol measure of health-related quality of life
ePRO: Electronic patient-reported outcomes
ITT: Intention to treat
KCL: King’s College London
MHE: myHealthE
NICE: The National Institute for Health and Care Excellence
ODD: Oppositional defiant disorder
PI: Principal Investigator
PPIE: Patient and Public Involvement and Engagment
PT: Parent Training
QALY: Quality-adjusted life year
RCT: Randomised controlled trial
REC: Research Ethics Committee
SDQ: Strengths and Difficulties Questionnaire
SLaM: South London and Maudsley NHS Foundation Trust
STEPS: Structured E-Parenting Support
PSC: Programme Steering Committee
WAU: Waitlist As Usual care
