# Supplementary material for: Online Parent Training for The Initial Management of ADHD referrals (OPTIMA): the protocol for a randomised controlled trial of a digital parenting intervention implemented to support parents and children on a treatment waitlist
Source: Trials. 2022 Dec 12;23:1003. doi: 10.1186/s13063-022-06952-z (PMC9744042; doi:10.1186/s13063-022-06952-z)
Supplement: Supplementary file 1 — Additional file 1. Full study protocol. [file 13063_2022_6952_MOESM1_ESM.pdf]

Online Parent Training for The Initial Management of ADHD Referrals: A two-arm parallel randomised controlled trial of a digital parenting intervention implemented on a treatment waitlist.

Protocol Short Title/ Acronym: OPTIMA RCT

Trial Identifiers

**ISRCTN:**	16523503		
**REC Number:**	21/NW/0319		
**UKCRN Number:**	CPMS ID: 50901		
**IRAS Number:**	303121		
**Protocol Version Number:**	2.2	Date:	01 July 2022

(Co) Sponsor(s)

**Name:**	King's College London	The South London and Maudsley NHS Foundation Trust (SLaM)
**Address:**	Room 5.31, James Clerk Maxwell Building, 57 Waterloo Road, London SE1 8WA	R&D Office (POO5), Room W1.09, IoPPN Main Building, King's College London, De Crespigny Park, London SE5 8AF
**Telephone:**	+44 (0)207 8483224	+44 (0)20 7848 0675
**Email:**	reza.razavi@kcl.ac.uk	slam-ioppn.research@kcl.ac.uk

Chief Investigator

**Name:**	Professor Edmund Sonuga-Barke
**Address:**	Department of Child & Adolescent Psychiatry, IoPPN, King's College London, 16 De Crespigny Park, London SE5 8AF
**Telephone:**	+44 (0)20 7848 0269
**Email:**	edmund.sonuga-barke@kcl.ac.uk

Name and address of Co-Investigator(s), Statistician, Therapy Service, Laboratories etc

**Name:**	Mrs Claire Ballard
**Position/ Role:**	Research Assistant
**Organisation:**	King's College London
**Email:**	claire.ballard@kcl.ac.uk

**Name:**	Professor Sarah Byford
**Position/ Role:**	Senior Trial Health Economist
**Organisation:**	King's College London
**Email:**	s.byford@kcl.ac.uk

**Name:**	Professor Samuele Cortese
**Position/ Role:**	Liaison between the University of Southampton and Solent NHS Trust
**Organisation:**	University of Southampton
**Email:**	samuele.cortese@gmail.com

**Name:**	Professor David Daley
**Position/ Role:**	Advisor on the STEPS programme.
**Organisation:**	University of Nottingham
**Email:**	david.daley@nottingham.ac.uk

**Name:**	Dr Johnny Downs
**Position/ Role:**	Leading the implementation of myHealthE across the trial sites.
**Organisation:**	King's College London
**Email:**	johnny.downs@kcl.ac.uk

**Name:**	Dr Blandine French
**Position/ Role:**	Research Assistant
**Organisation:**	University of Nottingham
**Email:**	blandine.french@nottingham.ac.uk

**Name:**	Professor Cristine Glazebrook
**Position/ Role:**	Joint lead for co-ordinating qualitative analyses.
**Organisation:**	University of Nottingham
**Email:**	Cris.Glazebrook@nottingham.ac.uk

**Name:**	Dr Kimberley Goldsmith
**Position/ Role:**	Senior Trial Statistician
**Organisation:**	King's College London
**Email:**	kimberley.goldsmith@kcl.ac.uk

**Name:**	Dr Charlotte Hall
**Position/ Role:**	Local oversight of the project at the Nottingham Trial Centre.
**Organisation:**	University of Nottingham
**Email:**	Charlotte.Hall@nottingham.ac.uk

**Name:**	Dr Ellen Hedstrom
**Position/ Role:**	Research Assistant
**Organisation:**	University of Southampton
**Email:**	E.E.Hedstrom@soton.ac.uk

**Name:**	Dr Zina Ibrahim
**Position/ Role:**	Expert in health informatics
**Organisation:**	King's College London
**Email:**	zina.ibrahim@kcl.ac.uk

**Name:**	Ms Lucinda Powell
**Position/ Role:**	Chair of the Public and Patient Involvement Committee
**Organisation:**	Member of the public
**Email:**	changingstatesofmind@gmail.com

**Name:**	Dr Kasia Kostyrka-Allchorne
**Position/ Role:**	Trial manager
**Organisation:**	King's College London
**Email:**	kasia.kostyrka-allchorne@kcl.ac.uk

**Name:**	Dr Hanna Kovshoff
**Position/ Role:**	Joint lead for co-ordinating qualitative analyses.
**Organisation:**	University of Southampton
**Email:**	H.Kovshoff@soton.ac.uk

**Name:**	Dr Jana Kreppner
**Position/ Role:**	Responsible for local oversight of the project at the Southampton Trial Centre.
**Organisation:**	University of Southampton
**Email:**	J.Kreppner@soton.ac.uk

**Name:**	Ms Nancy Lean
**Position/ Role:**	Research Administrator
**Organisation:**	King's College London
**Email:**	Nancy.lean@kcl.ac.uk

**Name:**	Mr Oliver Pearson
**Position/ Role:**	Trial statistician
**Organisation:**	King's College London
**Email:**	oliver.pearson@kcl.ac.uk

**Name:**	Professor Kapil Sayal
**Position/ Role:**	Liaison between the University of Nottingham and local clinical services.
**Organisation:**	University of Nottingham
**Email:**	Kapil.Sayal@nottingham.ac.uk

**Name:**	Dr James Shearer
**Position/ Role:**	Trial health economist
**Organisation:**	King’s College London
**Email:**	james.shearer@kcl.ac.uk

**Name:**	Professor Emily Simonoff
**Position/ Role:**	Local oversight of the project at South London Trial Centre and the liaison between SLaM and King’s College London.
**Organisation:**	King’s College London
**Email:**	Emily.simonoff@kcl.ac.uk

**Name:**	Professor Margaret Thompson
**Position/ Role:**	Advisor on the STEPS programme.
**Organisation:**	Solent NHS Foundation Trust
**Email:**	mt1@soton.ac.uk

**Name:**	Dr Lukasz Zalewski
**Position/ Role:**	Research platform engineer
**Organisation:**	King’s College London
**Email:**	lukasz.zalewski@kcl.ac.uk

1. Study Synopsis

**TITLE OF CLINICAL TRIAL:**	Online Parent Training for The Initial Management of ADHD Referrals: A two-arm parallel randomised controlled trial of a digital parenting intervention implemented on a treatment waitlist.
**Protocol Short Title/ Acronym:**	OPTIMA RCT
**Study Phase If Not Mentioned In Title:**	N/A
**Sponsor Name:**	King's College London/South London and Maudsley NHS Trust
**Chief Investigator:**	Professor Edmund Sonuga-Barke
**UKCRN Number:**	CPMS ID: 50901
**REC Number:**	21/NW/0319
**Medical Condition or Disease Under Investigation:**	Behavioural problems
**Purpose of Clinical Trial:**	To evaluate the effectiveness and cost effectiveness of a digital app (STEPS) designed to help parents of children with high levels of hyperactivity/impulsivity, inattention, and conduct problems, who are waiting to be assessed by Child and Adolescent Mental Health or Paediatric services.
**Primary Objective:**	To evaluate whether, in children screened positive for high levels of hyperactivity/impulsivity, inattention, and conduct problems, STEPS reduces the severity of behaviour problems rated by parents (primary outcome) measured at 3 months post-randomisation compared to Wait-As-Usual (WAU) care.
**Secondary Objective(s):**	1. To test for the maintenance of effects observed concerning primary outcome at 6-, 9- and 12-months post-randomisation.<br>2. To test whether STEPS reduces the severity of parent-rated hyperactivity/impulsivity and inattention at 3- and 12-month follow-up, as compared to WAU.<br>3. To test whether STEPS improves parenting (i.e., style, satisfaction and efficacy), increases child-parent closeness and reduces parenting-related strain at 3- and 12-month follow-up, as compared to WAU.<br>4. To establish the cost-effectiveness of STEPS compared to WAU at 3 months and 12-months post-randomisation with outcomes measured in terms of quality adjusted life years.<br>5. To use qualitative data from parents and clinicians to explore: how STEPS works; what the mechanisms of change are; and contextual factors influencing implementation of the app, in a way that is consistent with MRC guidelines for complex intervention process research.
**Exploratory objective(s):**	1. To examine whether STEPS reduces independently rated levels of child oppositionality and defiance expressed in speech during a period of task-focused parent-child interaction, as compared to WAU.<br>2. To examine whether STEPS reduces the severity of parent-rated emotional problems at 3- and 12-month follow-up, as compared to WAU.<br>3. To conduct an exploratory post-randomisation effect modifier analysis to examine whether app usage levels and patterns up to 3-months influence the effect of STEPS

	on the primary behaviour problems outcome at 3-months and the secondary behaviour problems outcome at 12-months.<br>4. To conduct an exploratory post-randomisation effect modifier analysis to see whether contact within clinical services outside of the service that the child had been referred to up to 3-months influences the effect of STEPS on the primary behaviour problems outcome at 3-months, and if such clinical contact up to 12-months influences the effect of STEPS on the behaviour problems secondary outcome effect at 12-months.<br>5. To conduct an exploratory analysis to see if STEPS reduces the likelihood of a clinical diagnosis being made and medication prescribed by the 12 months follow up.
**Trial Design:**	A two-arm superiority parallel randomised controlled trial with an internal pilot.
**Outcomes:**	*Primary:* Parent-reported mean child behaviour problems measured with the eight-item ODD subscale of the Swanson Nolan and Pelham Rating Scale (SNAP-IV).<br>*Secondary:*<br>1. Mean parent-rated child hyperactivity/impulsivity and inattention measured with the respective subscale of the SNAP-IV.<br>2. Parenting style measured with two subscales of The O'Leary Parenting Scale.<br>3. Parenting satisfaction and efficacy measured using the Parental Sense of Competence scale.<br>4. Parenting-related strain measured with the Caregiver Strain Questionnaire.<br>5. Child-parent closeness measured with a subscale from the Child-Parent Relationship Scale.<br>6. Children's and parents' health-related quality of life. The former will be measured with the Child Health Utility measure (CHU9D) and the latter with the EQ-5D-5L.<br>*Exploratory:*<br>1. Mean rating of child oppositional and defiant speech during an online parent-child drawing task, Etch-a-Sketch Online, measured with independent observer ratings on Childhood Oppositional and Defiance Speech Sample Scale.<br>2. Mean parent-rated child emotional problems measured with the respective subscale of the Strengths and Difficulties Questionnaire.<br>3. Whether children had received a diagnosis/been prescribed medication by 12 months follow up.
**Sample Size:**	352
**Summary of Eligibility Criteria:**	1. Parents of new referrals of children aged 5 to 11 years that passed the initial triage by the clinical service and have been accepted onto the assessment waiting list within that service and their referred child.<br>2. A routine positive screen for high levels of hyperactivity/impulsivity, inattention, and conduct problems using the hyperactivity (≥8) and conduct problems (≥4) subscale of the Strengths and Difficulties Questionnaire.<br>3. Access to a STEPS compatible digital device with access to the internet:<br>• iPhone with an operating system iOS 9.0 or later<br>• Android phone with an operating system 4.1 or later.
**Intervention (Description, frequency, details of delivery)**	Structured E-Parenting Support (STEPS) is a parenting support intervention delivered in the form of a mobile phone app. STEPS aims to help parents to be more effective and self-confident in managing child behavioural problems.<br>STEPS can be accessed through a smartphone. Parents can move through the content (steps) at their own pace and at any time of day. The order of the steps is fixed, although there is a degree of choice within each step. STEPS is an unguided intervention, which means there is no personal clinical support for parents progressing through STEPS. The content is delivered using short videos, audio clips and text.

				STEPS has one preparatory module, "Introduction", followed by eight separate intervention modules (steps). These steps must be followed in order. Each of the 8 steps is designed to take about 20 minutes if completed in one go. Parents will be asked to use the app regularly during the 3-month period.
			**Comparator Intervention:**	Wait-As-Usual (WAU) care
			**Maximum Duration Of Treatment Of A Subject:**	3 months
			**Version And Date Of Final Protocol:**	Version 2; 15 November 2021
			**Version And Date Of Protocol Amendments:**	Version 2.1; 14 January 2022<br>Version 2.2; 01 July 2022

2. Revision History

Document ID - (Document Title) revision X.Y	Description of changes from previous revision	Effective Date
V.2.1 15 November 2021	1. Push notifications sent from the STEPS app will be replaced with text messages also sent from the app (p. 19).<br>2. Telephone interviews will be recorded via Microsoft Teams and not audio capture software installed on a laptop (p. 32).	

V.2.2. 01 July 2022	1. Change to the character of an outcome: *Mean rating of child oppositional and defiant speech during an online parent-child drawing task, Etch-a-Sketch Online, measured with independent observer ratings on Childhood Oppositional and Defiance Speech Sample Scale* has been changed from secondary to exploratory outcome. A clear distinction has also been made between the secondary and exploratory outcomes.<br><br>*2.* Addition of a new exploratory outcome: *Mean parent-rated child emotional problems measured with the respective subscale of the Strengths and Difficulties Questionnaire.*<br><br>*3.* Changes to the study team:<br>- Lucinda Powell has replaced Christine Jarvis as the OPTIMA PPI Panel Chair.<br>- Oliver Pearson has replaced Walter Muruet-Gutierrez as the Trial Statistician.<br>- Dr Maddie Groom stepped down from her role as the interim co-lead for the Nottingham Trial Centre.<br><br>4. Correction of a typographical omission on p. 23. 10.3.8 Experience of parenting – T1 in now included in the subheading.<br><br>5. Changes to the amount of information collected by the STEPS app (p. 44). Following a recent update, STEPS no longer collects information about IP addresses and device IDs.<br><br>6. Addition of a definition of the study end date (p. 16).	

3. Glossary of terms

ADHD	Attention-Deficit/Hyperactivity Disorder
AE	Adverse Event
ANCOVA	Analysis of Covariance
AR	Adverse Reaction
ASD	Autism Spectrum Disorder
ASR	Annual Safety Report
CAMHS	Child & Adolescent Mental Health Services
CA-SUS	Child & Adolescent Service Use Schedule
CHU9D	Child Health Utility measure
CI	Chief Investigator
DMC	Data Monitoring Committee
EQ-5D-5L	EuroQol measure of health-related quality of life
ePRO	Electronic patient-reported outcomes
ICAN	Interactive CAMHS Assessment Network

ICF	Informed Consent Form
ITT	Intention to Treat
KCL	King's College London
MHE	myHealthE
NICE	The National Institute for Health and Care Excellence
ODD	Oppositional Defiant Disorder
Onboarding	Signing participants with the STEPS app
PI	Principal Investigator
PPI	Patient and Public Involvement
PT	Parent Training
QALY	Quality Adjusted Life Year
RCT	Randomised Controlled Trial
REC	Research Ethics Committee
SAE	Serious Adverse Event
SDQ	Strengths and Difficulties Questionnaire
SLaM	South London and Maudsley NHS Foundation Trust
STEPS	Structured E-Parenting Support
PSC	Programme Steering Committee
WAU	Waitlist As Usual care

1. STUDY SYNOPSIS **6**
2. REVISION HISTORY **8**
3. GLOSSARY OF TERMS **9**
4. BACKGROUND & RATIONALE **14**
5. TRIAL OBJECTIVES **16**
5.1 PRIMARY OBJECTIVE 16
5.2 SECONDARY OBJECTIVES 16
5.3 EXPLORATORY OBJECTIVES 16
6. STUDY DESIGN **17**
7. STUDY SETTING **17**
8. ELIGIBILITY CRITERIA **17**
8.1 INCLUSION CRITERIA 17
8.2 EXCLUSION CRITERIA 18
9. TRIAL INTERVENTION **18**
9.1 STEPS 18
9.1.1 STEPS content. *19*
9.1.2 STEPS structure *19*
9.2 FREQUENCY AND DURATION OF INTERVENTION 20
9.3 INTERVENTION RECORDS 20
9.4 COMPARATOR ARM: WAIT AS USUAL (WAU) 20
9.5 STRATEGIES TO INCREASE ENGAGEMENT WITH THE APP. 20
9.6 CONCOMITANT CARE 20
10. MEASURES AND OUTCOMES **20**
10.1 CHILD OUTCOMES 20
10.1.1 Parent-rated behaviour problems *21*
10.1.2 Parent-rated hyperactivity/impulsivity and inattention *21*
10.1.3 Parent-rated emotional problems *21*
10.1.4 Independent observer-rated behaviour problems. *21*
10.2 PARENT/FAMILY OUTCOMES 22
10.2.1 Parenting style: laxness *22*
10.2.2 Parenting style: over-reactivity *22*
10.2.3 Parenting satisfaction *22*
10.2.4 Parenting efficacy *22*
10.2.5 Parenting-related strain *23*
10.2.6 Closeness of the child-parent relationship *23*
10.3 OTHER MEASURES 23
10.3.1 Characterisation of the child's behaviour problems *23*
10.3.2 Autism spectrum disorder (ASD) symptoms *23*
10.3.3 Family characteristics and demographic measures *24*
10.3.4 App usage data *24*
10.3.5 Clinical diagnosis *24*
10.3.6 Pharmacological treatment *24*
10.3.7 Trial expectations *25*
10.3.8 Experience of parenting *25*

10.3.9 Adverse events ... *25*
10.4 ECONOMIC MEASURES ... 25
10.4.1 Resource-use data ... *25*
10.4.2 Child's health-related quality of life ... *26*
10.4.3 Parental health-related quality of life ... *26*
11. PROCESS EVALUATION ... 27
12. TIME SCHEDULE OF ENROLMENT AND ASSESSMENT ... 28
13. SAMPLE SIZE ... 28
14. RECRUITMENT ... 29
14.1 METHOD 1: MYHEALTHE PORTAL ... 29
14.2 METHOD 2: INTERACTIVE CAMHS ASSESSMENT NETWORK (ICAN) ... 30
14.3 METHOD 3: MANUAL REVIEW OF CURRENT REFERRALS ... 30
14.3.1 Consent for contact ... *30*
14.3.2 Clinic referral ... *31*
14.4 CLINICIAN INTERVIEWS RECRUITMENT ... 31
15. ALLOCATION ... 31
16. BLINDING ... 32
17. DATA COLLECTION ... 32
18. DATA MANAGEMENT ... 34
18.1 STUDY DATABASE ... 34
18.2 DATA STORAGE AND DATA TRANSFER ... 34
19. STATISTICAL ANALYSIS ... 35
19.1 CLINICAL EFFECTIVENESS ... 35
19.2 EXPLORATORY POST-RANDOMISATION EFFECT MODIFIER ANALYSIS ... 36
19.3 HEALTH ECONOMIC ANALYSIS ... 37
20. DATA MONITORING ... 38
20.1 PROGRAMME STEERING COMMITTEE ... 38
20.2 DATA MONITORING COMMITTEE ... 38
21. HARMS ... 39
21.1 SPECIFICATION, TIMING AND RECORDING OF SAFETY PARAMETERS ... 39
21.2 PROCEDURES FOR RECORDING AND REPORTING ADVERSE EVENTS ... 39
21.2.1 Definitions of adverse events. ... *39*
21.2.2 Adverse events reporting parameters ... *39*
21.3 EXPECTED ADVERSE EVENTS ... 41
21.4 PROGRESSION CRITERIA / STOPPING RULES ... 41
22. AUDITING ... 42
23. ETHICS & REGULATORY APPROVALS ... 42
24. PROTOCOL AMENDMENTS ... 43
25. CONSENT ... 43
25.1 PARENTS ... 43
25.2 CHILDREN ... 43
25.3 CLINICIANS ... 44
25.4 WITHDRAWAL OF PARTICIPANTS ... 44
26. CONFIDENTIALITY ... 45

27. PUBLICATION POLICY 45
28. INSURANCE / INDEMNITY 45
29. FINANCIAL ASPECTS 46
30. SIGNATURES 46
31. APPENDICES 46
APPENDIX 1 46
APPENDIX 2 48
APPENDIX 3 52
APPENDIX 4 54

The electronic version of this document is the latest version. It is responsibility of the individual to ensure that any paper material is the current version. Printed material is uncontrolled documentation.

4. Background & Rationale

Attention-deficit/hyperactivity disorder (ADHD) is a common neuro-developmental condition characterised by symptoms of inattention and/or impulsivity-hyperactivity (Dalrymple, Maxwell, Russell, & Duthie, 2019). It is associated with a range of academic, employment and social negative outcomes and impairment (Dalsgaard, Mortensen, Frydenberg, & Thomsen, 2013; Erskine et al., 2013; Fletcher, 2014; Moyá, Stringaris, Asherson, Sandberg, & Taylor, 2014; Polderman, Boomsma, Bartels, Verhulst, & Huizink, 2010). In 2012, annual ADHD-related health, social care and education costs in the UK were estimated at £670 million (Telford et al., 2013). These negative impacts are often driven by a broader pattern of behaviour problems (e.g., oppositional, disruptive, defiant and challenging behaviours) which are extremely common, in addition to the symptoms of inattention and hyperactivity/impulsivity themselves (Podolski & Nigg, 2001). Effective multi-modal treatments for ADHD exist but access to them in the UK is currently limited by budgetary constraints and there are long waiting lists for specialist expert assessment, diagnosis and treatment (The Children's Commissioner, 2016). In 2014, The House of Commons Health Committee reported that even maximum acceptable waiting times for complex cases of neuro-developmental disorders such as ADHD were already as long as 15 weeks in 2013 (Committee, 2014). In terms of actual waiting times, the Children's Commissioner Lightening Report in 2016 found up to 200 days delay to initial assessment (The Children's Commissioner, 2016). Prolonged waiting time may lead to families feeling unsupported during this stressful period and, importantly, to exacerbation of their difficulties, a decrease in motivation, poorer engagement and premature dropout with regard to future treatment (McGarry et al., 2008).

The goal of the *On-line Parent Training for the Initial Management of ADHD referrals* (OPTIMA) is to provide support for families for 3 months during the period between referral acceptance and the first full specialist clinical assessment. It focuses on supporting parents to better manage their children's co-occurring behaviour problems. These present a major challenge to parents - increasing levels of parenting stress (Theule, Wiener, Rogers, & Marton, 2011) and therefore the likelihood of mental health problems (Weitlauf, Vehorn, Taylor, & Warren, 2014). They contribute to coercive child-parent relationships that deteriorate over time (Johnston, 1996). It is the escalation of these behaviour problems to "crisis levels" that often lead parents with children who have ADHD-type problems to first seek professional help (Sayal, Taylor, & Beecham, 2003). For many parents finding a way to manage their child's disruptive and defiant behaviour is likely to be the most urgent treatment priority at the time of their initial referral.

Effective ways to help parents manage these behaviour problems already exist – these come broadly under the term Parent Training (PT; Dalrymple et al., 2019). A meta-analysis of randomized controlled trials (RCTs) shows that PT improves parent-child relations and reduces challenging behaviour in children with ADHD – both as seen from the parents' point of view and by independent observers who are blind to

treatment assignment (Daley et al., 2015; Groenman et al., 2021). PT is traditionally delivered face-to-face either in small groups or on a one-to-one basis which means that it is costly to provide and time-consuming to organise (Sonuga-Barke et al., 2018). PT is typically only offered after a full clinical assessment has been made and an ADHD diagnosis is given. This means that even in the best-case scenario parents can be left without support and guidance for long periods while waiting for the assessment process to take its course - risking the further deterioration of the parent-child relationship and escalation of their child's problems – with concomitant probable effects on parent and family wellbeing that this implies. However, the National Institute for Health and Care Excellence (NICE) recommends that, irrespective of the length of the waiting period before a full assessment is given, PT should be made available to families soon after their referral is made – allowing them to start addressing their children's behavioural difficulties early on.

In OPTIMA we address this challenge by employing a digital health intervention, a parenting app, designed to provide parents on the waitlist with support and advice so that they can better manage their children's behaviour. Three studies have examined digital PT for families with children showing ADHD-type problems (Breider, de Bildt, Nauta, Hoekstra, & van den Hoofdakker, 2019; DuPaul et al., 2018; Franke, Keown, & Sanders, 2020). Studies which compared online PT to a wait list condition showed significant reductions in children's problem behaviour (DuPaul et al., 2018) and an increase in parental competence, satisfaction in their parenting role, and maternal wellbeing (Franke et al., 2020). Though, it is worth noting that these two studies included children with elevated symptoms of ADHD rather than a diagnosis. Moreover, small sample sizes in these studies and the different intervention content mean that their results should be interpreted with caution.

The present study will be an RCT to evaluate a new app called STEPS (Structured E-Parenting Support). Inspired by the New Forest Parenting Programme (Sonuga-Barke, Daley, Thompson, Laver-Bradbury, & Weeks, 2001), STEPS was designed specifically to help parents manage the behaviour problems of their children referred with problems inattention and hyperactivity and impulsiveness (also described as ADHD-type) when used during the early post-referral/waitlist period. Key app objectives include: (i) increasing parents' knowledge of why their children behave in challenging ways, (ii) increasing parents' awareness of the importance of self-care and building their confidence as a parent, (iii) strengthening parent-child relationships and emphasising the importance of praise, (iv) facilitating effective communication patterns and (v) giving parents practical needs-based guidance and strategies allowing the more effective management of their children's behaviour. To keep costs to a minimum and to ensure that the STEPS app is a practical option that could be implemented within existing NHS resources, there is no personal clinical support for parents. However, a "Get help" facility in the app Settings will signposts participants to technical support. In addition, information on how to access the appropriate clinical service if required (CAMHS or GP) is provided within the Frequently Asked Questions (also available through the app Settings). For more information about STEPS, see Section 9 'Trial intervention'. In OPTIMA, STEPS will be evaluated, against wait-as-usual (WAU), using a two-arm parallel randomised controlled trial with an internal pilot. This study will evaluate the clinical and cost effectiveness of STEPS. We hypothesise that

The electronic version of this document is the latest version. It is responsibility of the individual to ensure that any paper material is the current version. Printed material is uncontrolled documentation.

STEPS will lead to improvements in children's behaviour as reported by parents in the short term (3 months post-randomisation) and these effects will persist over a 12-month follow-up period. These improvements will be accompanied by improved parenting and an increase in child-parent closeness, and reductions in parenting-related strain.

5. Trial Objectives

The overall objective of the trial is to evaluate a digital app (STEPS) designed to help parents of children with high levels of hyperactivity/impulsivity, inattention, and conduct problems, who are waiting to be assessed by Child and Adolescent Mental Health and Paediatric services.

5.1 Primary objective

To evaluate whether, in children screened positive for high levels of hyperactivity/impulsivity, inattention, and conduct problems, STEPS reduces the severity of behaviour problems (oppositionality and defiance) as rated by parents (primary outcome) measured at 3 months post-randomisation compared to WAU.

5.2 Secondary objectives

1. To test for the maintenance of effects observed regarding primary outcome at 6-, 9- and 12-months post-randomisation.
2. To test whether STEPS reduces the severity of parent-rated hyperactivity/impulsivity and inattention at 3- and 12-month follow-up, as compared to WAU.
3. To test whether STEPS improves parenting (i.e., style, satisfaction, and efficacy), increases child-parent closeness and reduces parenting-related strain at 3- and 12-month follow-up, as compared to WAU.
4. To establish the cost-effectiveness of STEPS compared to WAU at 3- and 12-months post-randomisation with outcomes measured in terms of quality adjusted life years.
5. To use qualitative data from parents and clinicians to explore: how STEPS works; what the mechanisms of change are; and contextual factors influencing implementation of the app, in a way that is consistent with MRC guidelines for complex intervention process research.

5.3 Exploratory objectives

1. To test whether STEPS reduces independently rated levels of child oppositionality and defiance expressed in speech during a period of task-focused parent-child interaction at 3- and 12-month follow-up, as compared to WAU.
2. To examine whether STEPS reduces the severity of parent-rated emotional problems at 3- and 12-month follow-up, as compared to WAU.

The electronic version of this document is the latest version. It is responsibility of the individual to ensure that any paper material is the current version. Printed material is uncontrolled documentation.

3. To conduct an exploratory post-randomisation effect modifier analysis to examine whether app usage levels and patterns up to 3-months influences the effect of STEPS on the primary behaviour problems outcome at 3-months and the secondary behaviour problems outcome at 12-months.
4. To conduct an exploratory post-randomisation effect modifier analysis to see whether contact within clinical services outside of the service that the child had been referred to up to 3-months influences the effect of STEPS on the primary behaviour problems outcome at 3-months, and if such clinical contact up to 12-months influences the effect of STEPS on the behaviour problems secondary outcome effect at 12-months.
5. To conduct an exploratory analysis to see if STEPS reduces the likelihood of a clinical diagnosis being made and medication prescribed by the 12 months follow up.

6. Study design

A two-arm superiority parallel randomised controlled trial with an internal pilot study (see below), with measures taken at baseline (T1), scheduled within one month before randomisation and then at three months (T2 primary outcome, primary timepoint), six months (T3), nine months (T4) and 12 months (T5) post-randomisation.

7. Study setting

The trial will be conducted remotely (using phone calls and digital methods), with researchers based in three Trial Centres: London, Southampton and Nottingham. Initially, recruitment for the OPTIMA study will take place across five sites delivering secondary (community paediatrics) and secondary/tertiary (child and adolescent mental health and behavioural support services) care and support for children's behavioural and mental health problems. These sites are in urban areas with catchment populations from a range of socioeconomic backgrounds. However, more sites will likely join during the project. A complete up-to-date list of study sites will be available on the OPTIMA website https://optimastudy.co.uk and will be reported in the main trial publication. Across all sites, participants will access the intervention (the STEPS app) using their own mobile device in their home setting. The end of the study will be the date of the last online questionnaire or ESO task completion or the final telephone follow-up of the last participant.

8. Eligibility criteria

8.1 Inclusion Criteria

- Parents of all new referrals of children aged 5 years 0 months to 11 years and 11 months that have been accepted onto the assessment waiting list and their referred children. A 'new referral' is defined as a child who has been on a waiting list for assessment for no longer than nine calendar months. This will be confirmed through screening medical records.
- Parents aged 18 years or older: confirmed through screening.

- Child screened positive for high levels of hyperactivity/impulsivity, inattention, and conduct problems: Child scored ≥8/10 on the hyperactivity/inattention and ≥4/10 on the conduct problems subscale of the SDQ, established through screening medical records. These cut-offs identify the top 10% of the population (Youth In Mind)
- English language competence: parent confirms through screening.
- Access to a STEPS compatible mobile device (iOS 9.0 or later; Android 4.1 or later) with access to the internet (i.e., mobile internet or wi-fi); parent confirms through screening.

8.2 Exclusion Criteria

- Child living under local authority care; confirmed through screening medical records.
- Child already received a clinical diagnosis of ADHD and/or received treatment for ADHD (pharmacological or non-pharmacological); confirmed either through screening medical records or discussion with parents.

If two children from the same family are referred during the trial at the same time and both meet inclusion criteria, then only the older of the two will be included. If they are referred at different times the first child will be included.

9. Trial Intervention

9.1 STEPS

Structured E-Parenting Support (STEPS) is a parenting support intervention delivered in the form of a digital application (app). STEPS has been designed to help parents to be more effective and self-confident in managing problem behaviour and so reduce levels of children’s oppositionality and defiance and conflict in the family. It is designed to be particularly helpful for the parents of children who are temperamentally more difficult to manage, such as those with attentional and impulse control problems (i.e., ADHD). It is aimed at supporting parents of primary school-aged children. STEPS was inspired by the New Forest Parenting Programme, a face-to-face parent training intervention. Its content has been shaped by the latest research about parenting and child behaviour as well as by many years of clinical experience.

STEPS can be accessed through a smartphone. Parents can move through the content, organised in modules (steps), at their own pace and at any time of day. The order of the modules is fixed, although there is a degree of choice within each module. STEPS is an unguided intervention, which means there is no personal clinical support for parents progressing through the app. The content is delivered mainly using short videos and audio clips. To register with the app, each parent will need their unique ID (provided by the study team), which will be used to link app usage data with the information obtained from other sources (i.e., questionnaires, medical records, interviews). After parent registers with the app, they will choose one of four ‘buddies’ – a parent character played by an actor, who will accompany them on their STEPS journey.

9.1.1 STEPS content.

STEPS has one preparatory module, "Introduction", and eight intervention modules (steps). These eight modules must be completed in the order presented in Table 1.

Table 1. The STEPS app modules' titles and aims.

MODULE TITLE	MODULE AIM
**1. Make a fresh start**	To encourage parents to see their child and themselves in a new, more positive way.
**2. Look after yourself**	To emphasize how important, it is for parents to find time for themselves and to make links with other parents.
**3. Get their cooperation**	To explain ways parents can communicate more effectively with their children
**4. Build their confidence**	To highlight how important, it is for parents to create situations in which they can praise their child
**5. Lead by example**	To help parents to think of ways they can avoid losing their temper with their children when they are being difficult.
**6. Guide & support them**	To show how parents can help their children navigate around difficult situations where they may find themselves getting upset.
**7. Give them structure**	To demonstrate how vital, it is that everyone signs up to and follows the house rules
**8. Reducing conflict**	To explain how using rewards and sanctions can promote better behaviour in children.

9.1.2 STEPS structure

Each module has a similar structure and includes the following common elements:

i) Aims: sets out the themes to be covered in a step presented by a parent Buddy.

ii) Science: provides a short and easy to understand video-description of the

evidence behind a step's message presented by a real-life expert.

iii) Examples: videos of parents (played by actors) discussing their parenting

experiences.

iv) Skills: audio presentations of specific skills in simple short sentences.

v) Reflections: a chance for parents to record, as text or audio, their experiences.

vi) Resources: downloadable aides and guides relating to each step.

There will be support for parents encountering technical difficulties. For any serious clinical concerns, parents will be provided with contact details for the clinical service to whom they were referred as well as direct contact details for crisis services.

9.2 Frequency and duration of intervention

Participants in the treatment arm will have access to STEPS for 3 months. STEPS is a self-guided intervention and the time needed to complete each module (step) will depend on the pace of the individual user. However, each of the eight modules are designed to take about 20 minutes if completed in one go. Completion of the two first modules: ‘Make a fresh start’ and ‘Look after yourself’ will constitute adherence to the intervention.

9.3 Intervention records

Parents’ use of the intervention will be recorded automatically within the app. Usage data will include the number of sessions started, the number of completed modules (steps) – that is the number of modules where the parent progressed through all the elements contained within that module (for a description of the elements contained within each module see 9.1.2. ‘STEPS structure’), the number of recorded reflections, the number of unlocked resources, the number of items added to favourites and the amount of time parents spent on watching videos or listening to audio skills.

9.4 Comparator Arm: Wait As Usual (WAU)

No alternative evidence-based intervention is currently recommended, available, or implemented in the United Kingdom during the post-referral waiting period for patients waiting for a child health services assessment. WAU, therefore, is the most appropriate comparator. Those randomized to WAU will receive STEPS after they have completed the 12-month follow-up. In the sites across our three centres, we do not expect any patients to receive their clinical assessment and initiate treatment within the first three months of randomisation (T2) or few - if any - patients to engage in self-initiated treatment during this period. Parents will not be stopped from initiating their own access to services over the 12 months of the trial. All treatments received will be carefully recorded throughout the trial through the Child and Adolescent Service Use Schedule (CA-SUS; Barrett et al., 2012) and the Assessment & Diagnosis form (see Appendix 1).

9.5 Strategies to increase engagement with the app.

The app has an attractive design and is easy to use. Its usability has been tested with a Patient and Public Involvement and Engagement panel. To promote engagement, automatically programmed digital reminders (text messages) will be sent from the app to participants reminding them to use STEPS. Levels of engagement will be monitored by evaluating the number of completed modules (steps) – data that are collected automatically by the app while it is being used.

9.6 Concomitant care

There will be no restrictions on concomitant care, which will be monitored carefully during the trial through the service use questionnaire (CA-SUS; Barrett et al., 2012).

10. Measures and outcomes

10.1 Child outcomes

10.1.1 Parent-rated behaviour problems

T1 covariate, sample description; mean difference at T2 as primary outcome; mean difference at T3, T4 and T5 as secondary outcomes.

This will be measured with the ODD subscale of the Swanson, Nolan, and Pelham Rating Scale – the MTA version (SNAP-IV; Swanson et al., 2001). The subscale consists of eight items that are rated on a 4-point scale (not at all, just a little, quite a bit, very much). The subscale score is obtained by averaging responses across the eight items (i.e., summing the items and dividing by 8). This SNAP-IV ODD subscale is a valid outcome measure for use in clinical trials (Hall et al., 2020).

Child disruptive and defiant behaviour was selected as the primary outcome because for many parents of children referred to clinical services, this is likely to be the most urgent treatment target at the time of their initial referral.

10.1.2 Parent-rated hyperactivity/impulsivity and inattention

T1 covariate, sample description, mean difference at T2 and T5 secondary outcomes.

This will be measured with the respective subscales of the SNAP-IV (Swanson et al., 2001). Each subscale of SNAP-IV consists of 9 items that are rated on a 4-point scale (not at all, just a little, quite a bit, very much). The subscale scores are obtained by averaging across the 9 items associated with the subscale (i.e., summing the items and dividing by 9) and can be further combined into a single ADHD scale score by deriving an average across the two subscales (i.e. summing the 18 items and dividing by 18) (Bussing et al., 2008; Hall et al., 2020) The SNAP-IV ADHD scale has confirmed validity for use as an outcome measure in clinical trials (Hall et al., 2020).

10.1.3 Parent-rated emotional problems

T1 covariate, sample description mean difference at T2 and T5 exploratory outcomes

This outcome will be measured by the respective SDQ subscale (Goodman, 1997). This subscale measures fearfulness, anxiety and low mood and consists of 5 positively phrased items rated on a 3-point scale (not true, somewhat true, and certainly true). Individual items' scores are summed to derive an overall emotional problems subscale score. Baseline scores (T1) will be extracted from the medical records, and we will used information from the SDQ completed nearest to the baseline assessment due date. T2 and T5 data will be collected online via the Sealed Envelope platform. We acknowledge that it may not be possible to collect T2 data in this way for all participants who are already enrolled in the trial. Therefore, if feasible, we may also extract T2 data from the medical records, if a participant completed the SDQ as part of the routine clinical follow-up within the T2 visit window.

Please note: in OPTIMA, parents will complete the full SDQ measure, as the questionnaire license holder does not permit a single subscale use.

10.1.4 Independent observer-rated behaviour problems.

T1 covariate, mean difference at T2 and T5 as exploratory outcomes.

Parents and children will jointly complete an online drawing task, Etch-a-Sketch Online (ESO; Oliver & Pike, 2021). It is a novel and validated online tool that allows remote observation of parent-child interactions at home. During the task lasting 5 minutes, parents and children will take turns drawing a simple picture on a mobile phone screen. The task will be audio recorded and the parent-child verbal exchange will be rated using the Child Oppositional and Defiance Speech Sample (CODS). This captures the level of four problematic aspects of child behaviour (i.e., being argumentative, defiant, easily annoyed, angry) by an independent observer (a member of the research team) using a 5-point scale (not at all, a little, moderately, very, extremely). The observers will be provided with a set of rating instructions and the rating process will be calibrated using a subset of 5 audio recordings before the rest of the rating is completed. Agreement between observers will be monitored on regular basis. The overall rating score will be derived by averaging across the 4 items corresponding to target behaviours (i.e., summing the items and dividing by 4).

10.2 Parent/family outcomes

10.2.1 Parenting style: laxness

T1 covariate, mean difference at T2 and T5 as secondary outcomes.

Laxness captures a lack of consistent responding with confirmed validity (Rhoades & O'Leary, 2007). This will be measured with a 5-item subscale from The O’Leary Parenting Scale or PS (Arnold, O'Leary, Wolff, & Acker, 1993). Probabilities of using specific discipline strategies in response to child misbehaviour are rated on a 7-point scale and are anchored by one effective and one ineffective response strategy. Responses to individual items are summed up to derive an overall subscale score.

10.2.2 Parenting style: over-reactivity

T1 covariate, mean difference at T2 and T5 secondary outcomes.

Over-reactivity will be measured with a 5-item subscale from The O’Leary Parenting Scale (Arnold et al., 1993), which is a valid outcome measure that captures overly emotional or harsh responding (Rhoades & O'Leary, 2007). This See ‘Parenting style: laxness’ for information about responding and scoring.

10.2.3 Parenting satisfaction

T1 covariate, mean difference at T2 and T5 as secondary outcomes

This outcome reflects domain-general parenting self-efficacy and satisfaction and will be measured with an 8-item subscale from the Parental Sense of Competence Scale (PSCS; Johnston & Mash, 1989). The items are positively and negatively framed, and parents make responses on a 6-point scale, with options ranging from “strongly disagree” to “strongly agree”. The subscale score is calculated for each participant by summing up individual item scores. This subscale has good validity (Ohan, Leung, & Johnston, 2000).

10.2.4 Parenting efficacy

T1 covariate, mean difference at T2 and T5 secondary outcomes

This outcome reflects the degree of anxiety, frustration, and motivation associated with the parental role and will be measured with an 7-item subscale from The PSCS (Johnston & Mash, 1989). For information about

calculating a subscale score see 10.2.4. ‘Parenting satisfaction’. The validity of this subscale remains unclear (Ohan et al., 2000).

10.2.5 Parenting-related strain

T1 covariate, mean difference at T2 and T5 as secondary outcomes.

This will be measured with the global score obtained on the Caregiver Strain Questionnaire (CGSQ; Brannan, Heflinger, & Bickman, 1997). CGSQ consists of 21-items with responses made on a 5-point Likert scale ranging from “not at all” to “very much”. Three indices are calculated for each participant by averaging individual item scores for the three subscales: Objective Strain (11 items), Subjective Internalised Strain (6 items) and Subjective Externalised Strain (4 items). Global Caregiver Strain Score is determined by calculating the sum of the three subscale scores. The scale is a reliable and valid measure of parenting-related strain (Brannan et al., 1997).

10.2.6 Closeness of the child-parent relationship

T1 covariate, mean difference at T2 and T5 as secondary outcomes.

This will be measured with the closeness subscale of the Child-Parent Relationship Scale – Short Form (CPRS-SF; Pianta, 1992). This 7-item subscale scale measures the extent to which parents feel that their relationship with a child is characterized by warmth, affection, and open communication that has good validity for measuring child-parent closeness (Driscoll & Pianta, 2011). Responses are made on 5-point Likert scale ranging from “definitely does not apply” to “definitely applies”. The items are summed to obtain a single subscale score.

10.3 Other measures

10.3.1 Characterisation of the child’s behaviour problems

T1 sample description.

This will be measured with the Child’s Challenging Behaviour Scale version 2 (CCBS; Bourke-Taylor, Pallant, & Law, 2014). The CCBS is a 9-item measure of challenging behaviours for children aged 5-18 years. Parents rate agreement with statements about their child behaviour on a 4-point scale ranging from “strongly agree” to “strongly disagree”, and the total score on the CCBS is calculated by summing the scores.

10.3.2 Autism spectrum disorder (ASD) symptoms

T1 sample description.

This will be measured with the Social Communication Questionnaire – lifetime version (SCQ; Berument, Rutter, Lord, Pickles, & Bailey, 1999). The SCQ is a 40-item screening measure for autism spectrum disorder and is validated to use for ages 4 years and above. Questions focus on behaviours that are likely to be observed by the primary caregiver and concern the following domains: reciprocal social interactions, language and communication and repetitive and stereotyped patterns of behaviours. The presence of autism-positive behaviour is coded as 1 and its absence as 0. The first question concerns the level of current language and is not included in the total score. For children with language all questions apply, and the

maximum score is 39; for children without language, the first 6 questions do not apply so the maximum score is 33 (language items are inapplicable).

10.3.3 Family characteristics and demographic measures

T1 sample description, process evaluation.

Parents will provide information about their child's age and sex, their own sex, their own and their child's ethnicity, parental education and employment and relationship status and whether there are other children with neurodevelopmental difficulties living in the household. We will estimate family socioeconomic status based on total household income, coded as < £16,000 | £16,000 - £29,999 | £30,000 - £59,999 | > £60,000 | Prefer not to say.

10.3.4 App usage data

During intervention period, intervention arm only. App use up to T2 as post-randomisation effect modifier for the primary SNAP ODD outcome at T2 and secondary ODD outcome at T5, process evaluation.

To establish intervention adherence, the number of completed modules will be measured (min = 0; max = 8), with completion of two modules constituting adherence to the intervention. Other collected app usage events will include: the number of started modules, the number of videos watched, and the time spent watching videos (in seconds), the number of audio clips listened to and the time spent listening to audio clips (in seconds), the number of reflections recorded, the number of items saved to favourites, and the number of accessed text resources. These will be used to provide descriptive information about app usage patterns. The format of the app usage data is being finalised as part of the feasibility study, so we are not yet able to specify the app usage variable(s) up to T2 that will be used as the effect modifier. Likely options include: the number of started modules or total time engaging with the app. We will aim to specify this in a future protocol amendment, and/or the statistical analysis plan.

10.3.5 Clinical diagnosis

Risk ratio at T5 as exploratory outcome.

Information about ADHD diagnosis will be extracted from medical records at 12-month follow-up and recorded on the Assessment & Diagnosis form (Appendix 1). In case it is not possible to access a child's medical record, information will be collected directly from a parent (via a phone call). A presence of ADHD diagnosis will be coded as 1 and absence as 0. In addition, if other diagnosis has been given, information about the type of diagnosis will be recorded for descriptive purposes.

10.3.6 Pharmacological treatment

Risk ratio at T5 as exploratory outcome.

Information about any pharmacological treatment for neurodevelopmental and mental health disorders will be extracted from medical records at 12-month follow-up and will be recorded on the Assessment & Diagnosis form (Appendix 1). In case it is not possible to access a child's medical record, information will be collected directly from a parent (via a phone call). A presence of pharmacological treatment will be coded as

1 and absence as 0. If pharmacological treatment has been prescribed, information about the name of the drug and dose will be recorded for descriptive purposes.

10.3.7 Trial expectations

T1, process evaluation.

Information about the participants expectations about parent training in general as well as the specific expectations about the STEPS app will be collected using a questionnaire developed specifically for the study (see Appendix 2). Parent will be asked to rate the statements on the Likert-type scale as well as to provide open-ended text responses.

10.3.8 Experience of parenting

T1, T2, T3, T4, T5, process evaluation

This will be measured with open-ended questions: “Think of a memorable interaction that you have had with your child within the last 24 hours. Tell us about that interaction. For example, what went well and why or what went badly and why? What might you do differently next time?”

10.3.9 Adverse events

T1, T2, T3, T4, T5, safety outcome.

This will be measured with the Medical and psychological events and difficulties questionnaire (MAPED), which has been developed for the study (see Appendix 3). Parents will be asked to report retrospectively any physical and/or mental health difficulties they or their child have experienced in the last three months. They will also be asked to report any difficulties they or their child have had with daily activities. These will be used to monitor safety and to provide descriptive information about adverse events experienced during the trial.

10.4 Economic measures

10.4.1 Resource-use data

T1, T2, T3, T4 and T5; (i) up to T2 and (ii) up toT5 contact with clinical services post-randomisation effect modifier for ODD at (i) T2 primary outcome, (ii) T5 secondary outcome.

This will be measured using the Child and Adolescent Service Use Schedule (CA-SUS; Barrett et al., 2012), a measure applied in a range of populations of young people with mental health problems. The CA-SUS collects information on the use of all hospital and community-based health services, social care and education services, service-provided accommodation (for example, Local Authority foster or residential care), and prescribed medications for mental health conditions.

The format of the variable(s) that will be used as the effect modifier has not been yet finalised. Likely options include: hospital or community appointments related to mental health, therapy or counselling, dietician/nutritionist appointment, neurofeedback, family support worker, voluntary helpline and parent training classes (e.g., Incredible Years Parent Training, Triple P, 1-2-3 Magic, Empowering Parents, Empowering Communities) or parent support groups. We will aim to specify this in a future protocol amendment, and/or the statistical analysis plan.

10.4.2 Child's health-related quality of life

T1, T2, T3, T4 and T5.

This will be measured with the Child Health Utility measure (CHU9D; Stevens, 2012). The CHU9D is a paediatric preference-based quality of life measure for use in healthcare resource allocation decision-making. The CHU9D has been designed for self-report by children aged 7 to 17, but with an interviewer's help, can also be used in children as young as 6-years-old (Canaway & Frew, 2013) and guidance is available from the developers for proxy completion by parents for children aged 5 and under. The current study, however, does not involve collecting data from children and thus the CHU9D will be completed by the parents using the proxy version of the measure. The questionnaire includes 9 items, each with a 5-level response category. Each item taps into a different domain of children's present functioning: worry, sadness, pain, tiredness, annoyance, school, sleep, daily routine and activities.

10.4.3 Parental health-related quality of life

T1, T2, T3, T4 and T5

This will be measured with the EQ-5D-5L (Herdman et al., 2011). The questionnaire is used to capture general health across five domains: mobility, self-care, usual activities, pain/discomfort and anxiety/depression. Each domain has five levels (no problem, slight problem, moderate problem, extreme problem and severe problem/unable to complete activities).

Table 2**.** OPTIMA RCT schedule of assessments.

	**STUDY PERIOD**										
	**Screening**	**Enrolment**	**Baseline**	**Randomisation**	**Intervention**			**Post-randomisation**			
**TIMEPOINT**	**-T_1**	**-T_1**	**T_1**					**T_2**	**T_3**	**T_4**	**T_5**
**Month**	**-1-0**	**-1-0**	**-1-0**	**0**	**0-1**	**1-2**	**2-3**	**3**	**6**	**9**	**12**
**SCREENING:**											
**Screening of medical records (HIC, age, LAC status)**	X										
**Screening by researcher (device, language)**	X										
**CONSENT**		X									
**Randomisation**				X							
**INTERVENTIONS:**											
STEPS					◆——	——	——◆				
WAIT AS USUAL					◆——	——	——◆				
**ASSESSMENTS:**											
Demographics			X								
SCQ			X								
CCBS			X								
SNAP-IV (ODD)			X					X	X	X	X
SNAP-IV (ADHD)			X					X			X
SDQ Emotional problems*			X					X			X
CPRS-SF (closeness subscale)			X					X			X
PS (laxness, overactivity)			X					X			X

PSCS			X					X			X
CGSQ			X					X			X
CHU9D			X					X	X	X	X
EQ-5D-5L			X					X	X	X	X
CA-SUS			X					X	X	X	X
ESO child assent			X					X			X
ESO			X					X			X
CODSS			X					X			X
APP USAGE:											
Number of completed steps					↔	↔	↔				
Other app usage events					↔	↔	↔				
Trial expectations			X								
Experience of parenting			X					X	X	X	X
Adverse events (MAPED)			X					X	X	X	X
Pharmacological treatment											X
Clinical diagnosis											X
Qualitative interviews								↔	↔	↔	↔

**T1 SDQ emotional subscale scores will be extracted from the child's medical records, T2 and T5 SDQ will be complete online via the Sealed Envelope platform.*

HIC = high levels of hyperactivity/impulsivity, inattention and conduct problems; LAC = child in local authority care; SCQ = The Social Communication Questionnaire; CCBS = Child's Challenging Behaviour Scale version 2; SNAP-IV = The Swanson, Nolan, and Pelham Rating Scale – the MTA version; ODD = Oppositional Defiant Disorder; ADHD = Attention/Deficit-Hyperactivity Disorder; SDQ = Strengths and Difficulties Questionnaire; CPRS-SF = The Child-Parent Relationship Scale- Short Form; PS = The O'Leary Parenting Scale; PSCS = The Parental Sense of Competence Scale; CGSQ = The Caregiver Strain Questionnaire; CHU9D = Child Health Utility; CA-SUS = The Child and Adolescent Service Use Schedule; MAPED = Medical and psychological events and difficulties questionnaire; CODSS = Child Oppositional and Defiance Speech Sample Scale; ESO = Etch-a-Sketch Online.

11. Process Evaluation

The process evaluation will follow the Medical Research Council guidelines for evaluating the implementation of complex interventions (Moore et al., 2015) and will be described in more detail in a separate protocol. Briefly, the process evaluation will use baseline and post-intervention quantitative trial data (e.g., app usage data, including counts and time) and qualitative data from semi-structured interviews with parents and clinicians (e.g., expectations, perceptions of impact, barriers to engagement) and textbox responses to open questions to explore the mechanisms for intervention implementation and impact.

All parents will be invited to respond to questions about: (1) their expectations of the trial (baseline) and (2) accounts of their experience of parenting during trial participation (T2 through to T5), see '10.3 Other measures'. This information will be collected using Red Pill online data collection platform. Detailed data on usage of the app will be automatically recorded for each participant in the STEPS arm. For details, see '10.3 Other measures'. Post-intervention, researchers will conduct remote (telephone, video call or email) in-depth, semi-structured interviews with a subgroup of parents in the intervention group (n ~ 50). Maximum variation, purposive sampling will be used to ensure that a full range of views and experiences are captured (taking account of demographic factors and levels of app engagement). In addition, telephone interviews

with clinicians (n ~ 10) will explore perceptions of the app and its perceived impact on preparing families for the formal clinical assessment. The interview schedules will be developed with Patient and Public Involvement (PPI) panel input. Interview data will be audio recorded and analysed using thematic analysis (Braun & Clarke, 2013), applying a framework approach (Gale et al 2013). As a thank you for taking part, all parents taking part in qualitative interviews will receive a £20 shopping voucher. There will be no incentives for clinicians.

12. Time schedule of enrolment and assessment

Recruitment into the trial will commence in January 2022 and will continue for 24 months. Final assessments (T5) are expected to be completed by 31 March 2025. The enrolment and group allocation are shown in Figure 1. The overall trial duration is 39 months. This is from first participant enrolled to the last follow-up assessment of the final participant is completed. Each individual participant will remain in the trial for approximately 12 months.

13. Sample size

A total of 352 parents and their children will be recruited into the study. This power calculation is based on projected effects at the primary endpoint (3 months post-randomisation). As behaviour problems measured with the ODD subscale of the SNAP-IV questionnaire (Swanson et al., 2001) is our primary outcome, we estimated the smallest difference of clinical importance between STEPS and WAU to be equivalent to an effect size d = 0.4 standard deviations based on the NICE guidance supporting the use of PT for the treatment of ODD or conduct disorder (Lundahl, Risser, & Lovejoy, 2006). A within trial drop-out rate of 25% is assumed. This is higher than in most previous trials of face-to-face PT programmes because of the unsupported nature of STEPS in OPTIMA. We will monitor drop-out rates during the internal pilot and, if necessary, recalculate the sample size if it is higher than the 25% estimated. Using Stata (version 14.0) command *sampsi,* ANCOVA analysis with a conservative zero correlation assumed between baseline and primary endpoint SNAP-IV ODD score, a two-sided test and an alpha of 0.05, we estimated that 176 individuals will be needed per trial arm (total n = 352) to provide 90% power to test the hypothesis that STEPS is superior to WAU.

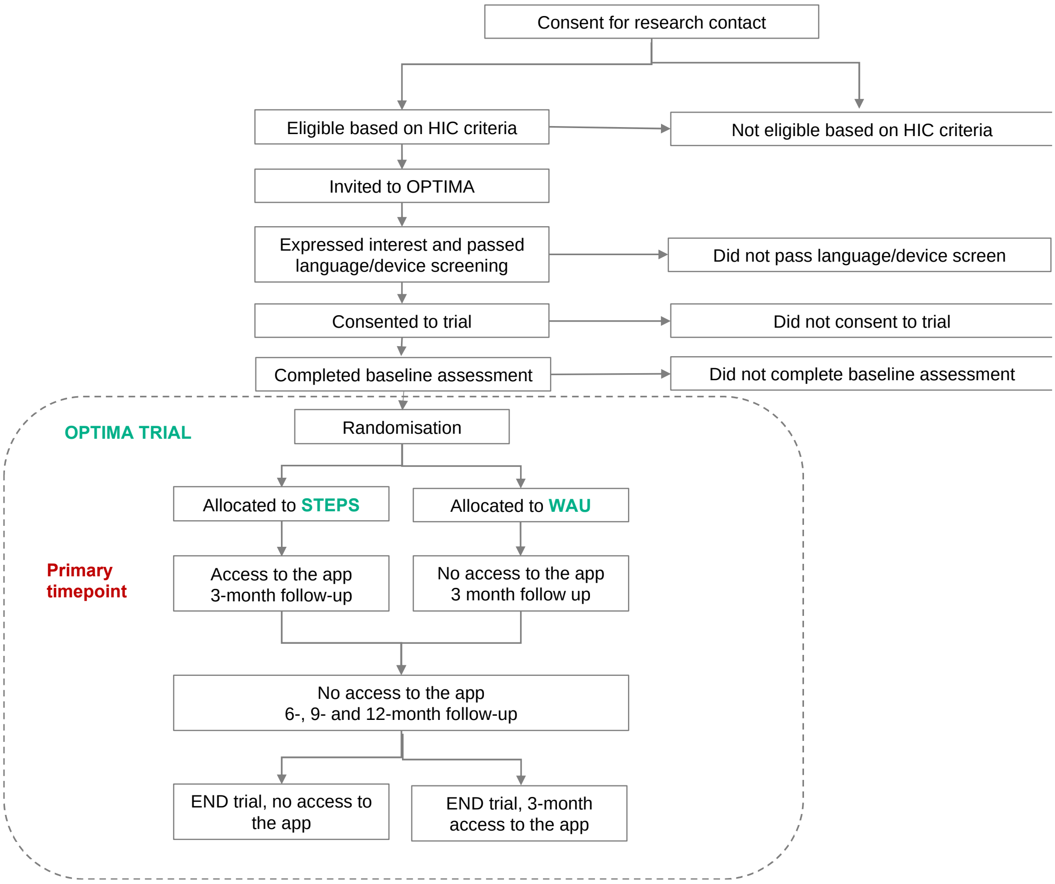

Figure 1. OPTIMA enrolment, group allocation and follow-up flowchart.

14. Recruitment

14.1 Method 1: myHealthE portal

In some of the participating organisations, clinical triage and rapid screening for high levels of hyperactivity/impulsivity, inattention, and conduct problems will occur as part of routine care using myHealthE (MHE) - a Caldicott Guardian approved, General Data Protection Regulation (GDPR) compliant, online portal for the automated screening of referred families using NHS CAMHS data (Ibrahim et al., 2015). MHE uses a secure text/email system through which primary caregivers are invited to register and complete validated clinical screening measures using an online portal. These are then automatically coded using standard algorithms to subsequently allow the research team to identify potentially eligible parents. MHE also seeks parents' permission to be contacted with invitations to take part in research studies; that is, provide consent-for-contact. By consenting to research contact, parents also give their permission for Trust-approved researchers to review their children's medical records to establish eligibility.

Authorised staff will regularly input the new referrals' details into MHE, which will initiate the mechanism for obtaining the initial consent for research contact and for obtaining the screening information. Research assistants will regularly log in to the MHE researcher portal (this portal only includes information about children whose parents gave consent-for-contact) to check for any new cases flagged up as 'eligible'. All

researchers conducting these reviews will have an appropriate letter of access or equivalent authorisation from the participating organisation, will be GCP trained, appropriately experienced, and have been delegated this responsibility by the CI on the delegation log. Research assistants will identify potential participants by reviewing cases flagged up as ‘eligible’ by MHE using a standard algorithm. To be flagged as ‘eligible’ the case must fulfil the following criteria: (1) a child is aged 5 years 0 months to 11 years 11 months, (2) a child screened positive for high levels of hyperactivity/impulsivity, inattention, and conduct problems on the SDQ; (3) a child is not under local authority care; and (4) a parent provided routine consent for research contact. The ‘eligible’ flag will be treated as a potential participating family.

In first instance, researchers will contact parents of eligible children by email with an invitation to take part in the study and a Parent Information Sheet. Non-responders will be followed up by phone, text message or post. If a parent expresses an interest in taking part (verbally, by email or by returning an expression of interest form), a researcher will arrange an initial telephone appointment with a parent to explain the details of the study, assess their English language competence and ascertain they have access to a STEPS compatible digital device. Assuming both the language screen and the device screen are positive parents will be asked to complete the consent form for the study online. There will be a minimum of 5 days between an invitation to take part and the initial telephone appointment.

14.2 Method 2: Interactive CAMHS Assessment Network (ICAN)

In North East London NHS Foundation Trust, routine outcome measures are collected through the Interactive CAMHS Assessment Network (ICAN). It is a digital application in which clinicians, young people, parents, caregivers, and teachers can provide routine clinical measures. ICAN provides useful feedback to clinicians to help track progress and data in real-time and is directly linked to the electronic patient record.

Parents of newly accepted referrals, who provide consent for research contact through ICAN, will be asked to complete the SDQ also through ICAN. This information will be then passed onto the research team, who will review the SDQ scores to establish eligibility. Parents of children who meet eligibility criteria will be approached by the researcher with an invitation to take part (by email, phone or post). Once a parent expresses an interest in taking part, a researcher will arrange a phone call to take the parent through the study details and the rest of the procedure will be identical to that described for participants identified through MHE (see ‘Method 1: myHealthE portal’).

14.3 Method 3: Manual review of current referrals

Manual review will be used if neither of digital methods (i.e., MHE or ICAN) are available within the participating organisation, or in case of any technical difficulties affecting the use of a digital platform.

14.3.1 Consent for contact

Members of the clinical teams in the participating organisations ask parents of patients if they are happy to be contacted about any relevant research. If they answer yes, this is recorded in their electronic health record

and gives Trust-employed Clinical Study Officers (CSOs) or researchers permission to screen their record for suitability and then make direct contact with the parent to invite them to the study.

The details of parents, who meet eligibility criteria and have given consent for contact will be securely transferred via encrypted email to the research team. Parents will be approached by the researcher with an invitation to take part (by email, phone, or post). Once a parent expresses an interest in taking part by responding to the invitation (by email, phone, or post), a researcher will arrange a phone call to take the parent through the study details and the rest of the procedure will be identical to that described for participants identified through MHE (see 'Method 1: myHealthE portal').

14.3.2 Clinic referral

Members of the clinical teams in the participating organisations will specifically discuss the study with the parent during a routine clinical contact and if they are interested and give verbal permission then pass their details to CSOs or researchers to take forward from there. This will act as their referral into the study. Information about the study, including a link to access the online information sheet, may also be emailed or posted by the clinical team to families (see 'Invitation letter/email wording'). Additionally, trust employed CSOs can be co-opted to work alongside the clinical team and make the initial contact with the parent (by phone) to invite them to take part in the study. The details of parents, who expressed interest in taking part in the trial will then be passed to the members of the research team via encrypted email and parents will be approached with an invitation to take part, as described above.

14.4 Clinician interviews recruitment

Clinicians taking part in the qualitative interviews (n ~ 10; see 11. Process evaluation) will be recruited from the services involved in the study. Service managers will be approached with a request to circulate the Clinician Information Sheet to the members of the team and the clinicians interested in taking part will be asked to contact the research team directly.

15. Allocation

Once the parent is enrolled in the study (i.e., after eligibility criteria are confirmed and informed consent has been obtained) and has completed the baseline assessments, they will be randomised to either the STEPS or WAU. Randomisation will be carried out online via a randomisation platform provided by Sealed Envelope in a 1:1 ratio and stratification by trial centre location (London, Nottingham, Southampton) using random permuted blocks procedure with varying block sizes. The researcher designated by CI (the trial manager or the trial administrator) will access the randomisation service, complete an on-screen form with participant details, inclusion and exclusion criteria and the randomisation allocation will be released. Randomisation system will use a unique identifying number. The web-based database system will send an automatic email confirmation of randomisation to unblinded researchers, who performed randomisation. The parent will be notified of the treatment allocation electronically (via email) or by phone by the researcher, who performed randomisation.

16. Blinding

Parents taking part in the study will remain unblinded throughout the trial; they will be informed about their group allocation after randomisation. Every effort will be taken to ensure that research assistants remain blinded (e.g., they will not have access to the app usage data). In addition, the risk of unblinding the research assistants will be minimal, as all follow-up data will be collected online using Red Pill Electronic Patient Outcome Forms (ePRO). The same number of automatic email/text prompts to complete the study measures will be sent to each participant, regardless of their arm allocation. To minimise the risk associated with unblinding during qualitative interviews, research assistants will conduct interviews with parents recruited from outside their local trial centre.

The senior statistician and senior health economist will not have access to the randomisation list or ePRO forms at any point in the trial to remain fully blinded until review of the first draft of the statistical/health economic reports for checking, when they will become fully unblinded. Similarly, the Chief Investigator and Principal Investigators in each trial centre will remain fully blinded until they review the finalised statistical report, when they will become fully unblinded. The junior statistician and junior health economist will be partially blinded until sign off of the statistical and health economic analysis plans, after which they will be fully unblinded so they can inspect and utilise app usage/therapy-related data. The trial manager and the trial administrators will be unblinded. The only individuals that will be able to summarise/see data by arm prior to the review of the statistical report are the junior statistician, junior health economist and the members of the data monitoring committee. We will presume that the data monitoring committee will remain partially blinded and will prepare the closed report accordingly. No serious harms associated with taking part in the intervention are expected, therefore a formal procedure for unblinding any blinded staff during the study is not needed.

17. Data collection

All OPTIMA trial data will be collected remotely: online, by phone and via video/audio chat. For each participant, assessments will take place over the period of 12 months and will be conducted at five timepoints as described above [baseline (T1), T2, T3, T4, T5]. The assessment window will cover a period of six weeks and will begin two weeks before the scheduled assessment and will close four weeks after the scheduled assessment. As a thank you for taking part, parents will receive shopping vouchers: £10 at baseline, £25 voucher at T2 and £10 voucher after completing T3, T4 and T5 assessments. Children will receive a £5 book voucher and a certificate for each completed task. The vouchers will be emailed to participants.
Four categories of data will be collected in the study: (1) participants' reports on the outcome measures and information about adverse events; (2) a speech sample audio-recorded during a remote parent-child drawing task (ESO); (3) the STEPS app usage data (for the intervention group only) and (3) video and audio recordings of qualitative interviews.

(1) Outcome data and information about adverse events will be collected online using Red Pill – a secure electronic data capture and management system provided by Sealed Envelope or over the phone by a trained researcher.
In first instance, participants will be notified by email and/or a text message when a scheduled assessment requires them to complete the questionnaires online using subject entered forms (ePRO) system provided by Red Pill. They will be able to complete the questionnaires using the browser on their computer or phone. Researchers will be able to track questionnaire completion within Red Pill using a display listing both complete and incomplete questionnaires for each participant. Up to three emails/text messages will be sent as reminders to complete online forms. Non-responders will be followed up with phone calls until the end of the assessment window. Through the information sheet, participants will be informed that, if they wish, they can also complete the questionnaires or provide information about adverse events over the phone. If they opt to provide information over the phone, a research assistant will arrange a convenient time to speak to the participant and will enter data onto either a paper form and later transfer the data into the study data base or directly onto an electronic form within Red Pill database during a phone call with a participant. Data entered into the Red Pill database will be de-identified.

(2) A 5-minute speech sample will be recorded during an online parent-child interaction drawing task via Microsoft Teams (ESO). During the task, participants will be identified by their unique ID number. They will also be asked to turn their camera off. The recorded sample will be rated by a trained researcher using Child Oppositional and Defiance Speech Sample Scale (CODSS) and observer's rating scores will be entered onto an electronic form within the Red Pill database.

(3) All app usage data will be collected within Google Firebase and will be stored with Google servers. Usage data held within Google Firebase will be of non-identifiable nature and will be stored against the study ID. Therefore, the risk of harm to participants following a potential privacy breach is low. Access to the STEPS Google Firebase account will be restricted to authorised users in the research team and external staff responsible for the app maintenance and support.

(4) Video and audio files. Whenever possible remote interviews will be conducted and recorded via Microsoft Teams. In case it is not possible to conduct an interview via Teams, participants will be given an option to take part in a telephone interview, which will also be recorded with Microsoft Teams. During remote interviews (via Microsoft Teams or telephone) participants will be identified by their unique ID number. Alternatively, a researcher will email a participant a list of questions, and they will be asked to respond by providing responses in text form. For interviews conducted by email, participants will be advised that security cannot be guaranteed when communicating by email using a non-secure email address and that it is not possible to maintain anonymity during data collection, but that any identifying information (e.g., their name and email address) will be redacted from the text and their de-identified data will be stored using a study ID.

18. Data management

18.1 Study database

The main trial database for the study will be provided by Sealed Envelope, which will ensure there are robust processes for quality management, security and back up of data https://www.sealedenvelope.com/security/. Database services and support and electronic data capture will be delivered through a service level agreement between Sealed Envelope and KCL. Access to the database and electronic data capture forms will be restricted by user identifiers and passwords to a limited number of researchers (i.e., trial manager and trial administrator, junior statistician, research assistants). Blinded researchers will not be able to view randomisation information within the database.

Sealed Envelope uses Red Pill- an online application for collecting and managing case report form (CRF) data on participants recruited to a clinical trial or other research studies. In this study, Red Pill will be used to record data collected offline (e.g., phone or medical records) as well as collect data directly from parents themselves [electronic patient reported outcomes (ePRO)].

The system used in the OPTIMA trial will be specifically configured for the study.

18.2 Data storage and data transfer

Handling of all data collected in the OPTIMA trial is described in detail in the OPTIMA Data Management Plan. In brief:

Data held within Red Pill database: Extracts of data will be downloaded by a junior trial statistician in plain text comma separated value format (.csv files) or Stata fixed format with a dictionary. Data will be retained within Red Pill database for three months after the database lock. After this time, all data files will be downloaded and stored within KCL SharePoint site and the Red Pill database will be deleted.

STEPS usage data: Extracts of the app usage data will be downloaded by researchers in plain text comma separated value format (.csv files). After data collection has been completed, all usage data will be downloaded onto KCL SharePoint site. The app-related Google Firebase account and all the data contained within the STEPS account will be deleted after the analysis is completed and the results are published in a peer-review journal.

Audio recordings of the speech sample during the ESO task recorded via Microsoft Teams will be initially stored within Microsoft Stream and access to Stream will be restricted by user identifiers and passwords. After a session, audio recordings will be downloaded onto a KCL OneDrive for Business and the files will be deleted from Microsoft Stream. The original recordings will be deleted from OneDrive for Business after transcription and coding have been completed.

Qualitative interviews: Consent forms completed by clinicians will be downloaded from Qualtrics and stored in electronic form on a KCL OneDrive for Business and SharePoint location, which is a web-based, Microsoft Office integrated collaborative platform. Video and audio recordings from the qualitative

The electronic version of this document is the latest version. It is responsibility of the individual to ensure that any paper material is the current version. Printed material is uncontrolled documentation.

interviews recorded via Microsoft Teams will be initially stored within Microsoft Stream and access to Stream will be restricted by user identifiers and passwords. Video and audio recordings and automatically generated transcripts will be downloaded onto a KCL OneDrive for Business and the files will be deleted from Microsoft Stream. The automatically generated transcripts will be checked by the members of the research team against the original recording and edited to remove any identifiable information. Interviews provided via email responses will be downloaded onto a KCL OneDrive for Business and de-identified. All de-identified transcripts and email responses will be stored in electronic form on a KCL OneDrive for Business and SharePoint location. The original recordings or emails will be deleted from OneDrive for Business after transcription.

Paper forms: Paper forms will be stored in the locked cabinet in the Trial Manager's office located in the Department of Child & Adolescent Psychiatry at King's College London.

Only de-identified data files will be used for analyses. The data extracted from the Red Pill database, the STEPS app usage data and qualitative interviews data will be linked using the study ID. All data files will be stored on King's College SharePoint location for the period of 7 years. The data stored on SharePoint are encrypted and access to the data will be restricted only to those who need to have it. All databases will be registered in the King's Data Protection Register (KDPR). After 7 years, personal data will be securely destroyed, and fully anonymised research data will be transferred to King's College Research Data Management or other suitable repository for long-term storage and stored for 13 years (overall, data will be held for 20 years).

19. Statistical analysis

19.1 Clinical effectiveness

Does STEPS reduce the average levels of *parent-rated* child behaviour problems in children screened positive for high levels of hyperactivity/impulsivity, inattention, and conduct problems, measured over a three- (primary outcome and timepoint), six-, nine- and 12-month (secondary outcomes) period, when compared to Wait-As-Usual (WAU)?

Does STEPS reduce the average levels of parent-rated child hyperactivity/impulsivity and inattention screened measured over a three- and 12-month (secondary outcomes) period, when compared to WAU?

Does STEPS improve the average levels of: parenting style, satisfaction and efficacy, and child-parent closeness; and reduce parental strain (additional secondary outcomes) in parents of children screened positive for high levels of hyperactivity/impulsivity, inattention, and conduct problems, measured over a three- and 12-month period, when compared to WAU?

EXPLORATORY 1: Does STEPS reduce the average level of children's oppositional and defiant speech measured by CODSS during an online child-parent interaction task (ESO) at 3- and 12-months post-randomisation, as compared to WAU?

EXPLORATORY 2: Does STEPS reduce the average level of children's emotional problems measured at 3- and 12- months post-randomisation, as compared to WAU?

EXPLORATORY 3: Does STEPS reduce the reduces the likelihood of a clinical diagnosis being made and medication prescribed by the 12 months follow up, as compared to WAU?

The main analysis will follow the intention to treat (ITT) principle as much as possible (Sullivan et al., 2018; White et al., 2012). A mixed effects linear analysis of covariance (ANCOVA) model with repeated measures will be used with the SNAP-IV ODD scores at three (primary outcome), six, nine and 12 months (secondary outcomes) post-randomisation as the dependent variables, and intervention group, time point, intervention group by time point interaction, baseline SNAP-IV ODD score, and the trial centre stratification variable as independent variables, with the interaction term used to extract the primary and secondary outcome STEPS vs WAU mean differences and associated 95% confidence intervals at the four time points. Similar statistical models will be used to test for intervention effects on the other continuous secondary and exploratory outcome scale scores (average levels of directly observer-rated child behaviour problems (ESO), parent-rated hyperactivity/impulsivity and inattention, parenting style, satisfaction and efficacy, child-parent closeness and parenting-related strain) with the scores extracted at the timepoints listed in section 10 'Measures and outcomes'. For the binary exploratory diagnosis and medication outcomes we will use modified Poisson regression with robust standard errors to estimate STEPS vs WAU relative risks (and associated 95% confidence intervals) for getting a diagnosis and being prescribed medication (Zou, 2004).
In addition to ITT analysis, we will undertake a complier average causal effect (CACE) sensitivity analysis (Angrist, Imbens, & Rubin, 1996; Dunn et al., 2015) on the ODD outcome primary outcome at 3 months (T2) and ODD secondary outcome at 12 months (T5), to estimate treatment effects in those who completed at least two modules of the STEPS app (i.e., those that complied with the intervention).

19.2 Exploratory post-randomisation effect modifier analysis

Do app usage levels and patterns up to 3-months influence the effect of STEPS on the primary behaviour problems outcome at 3-months and the secondary behaviour problems outcome at 12-months?

Does contact within clinical services outside of the service that the child had been referred to up to 3-months influences the effect of STEPS on the primary behaviour problems outcome at 3-months?

Does contact within clinical services outside of the service that the child had been referred to up to 12-months influences the effect of STEPS on the behaviour problems secondary outcome effect at 12-months.

Two exploratory post-randomisation effect modification analyses will be conducted. First, we will analyse the impact of the app usage (likely in terms of number of completed modules or total time spent in the app) collected up to T2 on the effects of STEPS on the primary ODD outcome measured at 3 months post-randomisation (T2) and the secondary ODD outcome measured at 12-months (T5). Second, the impact of clinical contact/care: up to 3 months (T2) on the effects of the STEPS app on the ODD primary outcome at 3 months, and up to 12 months (T5) on the effects of the STEPS app on the ODD secondary outcome at 12 months. Clinical contact/care will include self-initiation of treatment during the trial up to the two specified time points. The clinical care variables used as post-randomisation effect modifier will be extracted from the service use form (the CA-SUS; see section 10.4.1 ‘Resource use data’) and will likely be somewhat post hoc as we are not currently sure which variables are most appropriate; this approach will be made clear to the reader in the publication. We plan to use appropriate causal methods for post-randomisation variables to explore whether the effects of interventions differ by these variables, such as principal stratification analysis (Dunn et al, 2015).

Up to date versions of either the Stata or R software programs will be used for analysis. Further detail of the analyses described in this section will be provided in a statistical analysis plan.

19.3 Health economic analysis

Is STEPS cost- effective compared to WAU over the 3-month and 12-month follow-up periods?

The economic evaluation will adopt the NHS/personal social services perspective preferred by NICE, but will also include education-based services, given the age of the population. Resource-use data collected using the CA-SUS will be costed using nationally applicable unit costs (e.g., PSSRU Unit Costs of Health and Social Care, NHS Reference Costs, BNF for medications). The STEPS app will be costed in consultation with the system developers.

The primary economic evaluation will be a cost-utility analysis carried out at 3-months post-randomisation with outcomes expressed in terms of quality adjusted life years (QALYs), using the CHU9D proxy completed by a parent. Secondary economic analyses will include: (i) a cost-utility analysis at 12-months post-randomisation to assess cost-effectiveness subsequent to formal assessment and treatment; (ii) a cost-effectiveness analysis undertaken at both 3-months and 12-months using the primary clinical measure of outcome (SNAP-IV ODD score) and (iii) a cost-utility analysis undertaken at both 3-months and 12-months combining QALYs for both the young person (using the CHU9D) and the primary parent/caregiver (using the EQ-5D-5L). Appropriate sensitivity analyses will be carried out, dependent on any assumptions made in relation to the costing of the intervention, the method of measurement of outcomes or the approach to combining QALYs for young people and their primary carer.

Costs and outcomes will be compared at the 3-month and 12-month follow-up points and presented as mean values by trial arm with standard deviations. Mean differences in costs and 95% confidence intervals will be obtained by non-parametric bootstrap regressions to account for the non-normal distribution commonly found in economic data. To provide more relevant treatment-effect estimates, analyses will include adjustment for baseline covariates (Assmann, Pocock, Enos, & Kasten, 2000), which will be pre-specified and in line with the clinical analyses. Cost-effectiveness will be assessed using the net benefit approach following standard approaches (Briggs, Wonderling, & Mooney, 1997). A joint distribution of incremental mean costs and effects for the two groups will be generated using non-parametric bootstrapping to explore the probability that each of the treatments is the optimal choice, subject to a range of possible maximum values (ceiling ratio) that a decision-maker might be willing to pay for an additional QALY (or unit improvement in behaviour problems). Cost-effectiveness acceptability curves will be presented by plotting these probabilities for a range of possible values of the ceiling ratio (Fenwick & Byford, 2005). These curves are a recommended decision-making approach to dealing with the uncertainty that exists around the estimates of expected costs and expected effects associated with the interventions under investigation and uncertainty regarding the maximum cost-effectiveness ratio that a decision-maker would consider acceptable. We will additionally undertake a sensitivity analysis involving the extrapolation of costs and benefits into the future if the trial results suggest this would be worthwhile and the OPTIMA Data Monitoring Committee are in agreement.

20. Data monitoring

20.1 Programme Steering Committee

The Programme Steering Committee (PSC), a body independent of the research team will provide formal oversight and expert advice for the overall OPTIMA programme, which includes an oversight over the present trial. Its role is to ensure that the trial is conducted in a rigorous and timely manner and consider any proposed changes to the agreed programme of research. The PSC consists of an independent chair, statistician, digital health expert, digital mental health interventions expert, two parents of a child with ADHD, and health economics expert. The PSC will meet approximately every six months, with one meeting coinciding with the end of the internal pilot, to provide advice to the funder whether the trial should continue.

20.2 Data Monitoring Committee

The Data Monitoring Committee (DMC) is an independent body of experts that has been established to monitor the quality of trial data and the safety of participants. The DMC will have access to unblinded data if they wish. The DMC will be responsible for monitoring the overall conduct of the study, including recruitment, protocol compliance, accuracy and completeness of data collection. Based on this information, the DMC will make recommendations to the PSC, the Funder and the Sponsor on whether the study should continue or whether there are any ethical or safety reasons why the study should be modified or terminated. Any key changes to the study design and methodology will be reviewed by the DMC. The members of the DMC are completely independent of the trial and consist of an independent chair, statistician, and clinical

expert. The DMC will meet approximately every six months, shortly before a PSC meeting. One of the meetings will coincide with the end of the internal pilot, to determine whether the trial should continue. The DMC and the PSC will be given a charter to outline their tasks and responsibilities.

21. Harms

21.1 Specification, Timing and Recording of Safety Parameters

Please see section 21.2 below for adverse event definitions and reporting parameters. The risk of participants experiencing any adverse events during this trial as a result of using STEPS is very low. Adverse events concerning parental or child physical and mental health will be monitored throughout the trial. Any physical or mental health difficulties spontaneously disclosed by a parent in their communication with researchers will be entered into the Red Pill database using the OPTIMA adverse events form created specifically for the study (see Appendix 4). Moreover, at each timepoint, participants will be asked to complete a formal questionnaire on adverse events (Medical and psychological events and difficulties questionnaire) that happened to them and their child. This information will be collected online using ePRO. The forms will be reviewed regularly, and all adverse events will be recorded on the OPTIMA adverse events form in the Red Pill database. All participants that experience a serious adverse event will be followed-up by the researchers, who have completed Level 2 safeguarding training, until the event is resolved. Where necessary, the participant’s clinical service which accepted the referral will be informed about the event.

21.2 Procedures for Recording and Reporting Adverse Events

21.2.1 Definitions of adverse events.

Adverse Event (AE): Any untoward medical occurrence in a subject to whom a therapy has been administered including occurrences which are not necessarily caused by or related to that therapy.

Adverse Reaction (AR): Any untoward and unintended response in a subject to a therapy which is related to any duration of therapy administered to that subject.

Unexpected Adverse Reaction (UAR): An adverse reaction the nature and severity of which is not consistent with the information known about the therapy in question in the view of the investigator

Suspected Unexpected Serious Adverse Reactions (SUSAR): Any serious adverse events that is deemed to be related to the trial intervention and unexpected (not listed in the protocol as an expected side effect of the intervention).

In non-CTIMPs, a serious adverse event (SAE) is defined as an untoward occurrence that:
(a) results in death;
(b) is life-threatening;
(c) requires hospitalisation or prolongation of existing hospitalisation;
(d) results in persistent or significant disability or incapacity;
(e) consists of a congenital anomaly or birth defect; or
(f) is otherwise considered medically significant by the investigator**.**

21.2.2 Adverse events reporting parameters

An SAE occurring to a research participant should be reported to the main REC, where in the opinion of the Chief Investigator (CI) the event was:

- Related – that is, it resulted from administration of any of the research procedures, and
- Unexpected – that is, the type of event is not listed in the protocol as an expected occurrence.

All SAEs, SARs and SUSARs (excepting those specified in this protocol as not requiring reporting) should be reported immediately to the Chief Investigator and to the Sponsor.

Reports of Serious Adverse Events (SAEs) that are **related** to the study (i.e., they resulted from administration of any of the research procedures) and **unexpected** (i.e., not listed in the protocol as an expected occurrence should be submitted to the REC using the Non-CTIMP safety report to REC form.

These should be sent within 15 days of the Chief Investigator becoming aware of the event. Reports of SAEs in double-blind trials should be unblinded. There is no requirement for annual safety reports in addition to the information provided through the annual progress report. More detailed description of adverse events reporting parameters is included in Table 3.

Table 3. Adverse events reporting parameters.

	Who	When	How	To Whom
**SAE**	Chief Investigator	Within 15 days of CI becoming aware of the event	SAE Report form for Non-CTIMPs, available from NRES website.	Main REC with a copy to the sponsor
**Urgent Safety Measures**	Chief Investigator	Immediately<br><br>Within 3 days	By phone<br><br>Notice in writing setting out reasons for the urgent safety measures and the plan for future action.	Main REC<br><br>Main REC with a copy sent to the sponsor. The MREC will acknowledge this within 30 days of receipt.
**Progress Reports**	Chief Investigator	Annually (starting 12 months after the date of favourable opinion)	Annual Progress Report Form (non-CTIMPs) available from the NRES website	Main REC with a copy to the sponsor
**Declaration of the conclusion or early termination of the study**	Chief Investigator	Within 90 days (conclusion)<br>Within 15 days (early termination)<br>*The end of study should be defined in the protocol*	End of Study Declaration form available from the NRES website	Main REC with a copy to the sponsor
**Summary of final Report**	Chief Investigator	Within one year of conclusion of the Research	No Standard Format<br>However, the following Information should be included:<br>Where the study has met its objectives, the main findings and arrangements for publication or dissemination including feedback to subjects	Main REC with a copy to be sent to the sponsor

All completed adverse event questionnaires will be reviewed by the research team to identify any Suspected Unexpected Serious Adverse Reactions (SUSAR). If an event meets the definition of a SUSAR (see box below) then the member of staff to whom the event was disclosed must inform Chief Investigator within 24 hours of becoming aware of the serious adverse event and provide all necessary information. Each case will be assessed by the Chief Investigator and team clinical expert for seriousness, expectedness and relatedness to the study. The CI will then take appropriate action, which may include halting the study and informing the Sponsor of such action. If the event is deemed serious, unexpected and related to the study intervention they shall inform the REC using the reporting form found on the HRA web page within 15 days of knowledge of the event, and include the co-Sponsors (King's College London and The South London and Maudsley NHS Foundation Trust) in all communications. They shall, within a further eight days send any follow-up information and reports to the REC and make any amendments as required to the study protocol and inform the REC as required.

All SUSARs will be recorded and closely monitored by the research team until resolution, stabilisation, or until it has been shown that the study intervention is not the cause.

Any participant who experiences a SUSAR may be withdrawn from the study at the discretion of the CI and the Sponsor. Every withdrawal will be examined by the DMC. The DMC will be made aware of all serious adverse reactions/SUSARs at their regular meetings.

The CI will have responsibility for informing the other sites of the SUSAR and any relevant safety information, which may be delegated to the Trial Manager. The CI will also inform the local clinical care team (CAMHS or Community Paediatrics) or GP under which the participant is routinely seen for of any SUSARs (details of which are obtained at screening/referral).

Events categorised in the protocol as an expected (see 20.3), do not need to be reported.

21.3 Expected adverse events

The following list describes expected adverse events. These events will be reported in the main publication, but not to the REC, unless they meet the serious adverse reaction criteria, in which case they will be reported as described above.

School/community:

- Increased child refusal to go to school/community activities.
- Exclusion from school/community activities.

Family dynamics:

- Deterioration in child behaviour (including self-harm) or wellbeing.
- Deterioration in sibling wellbeing (including self-harm).
- Increased family discord.
- Breakdown in family structure.
- Social work involvement or child protection concerns.

Parent wellbeing:

- Increased depressed mood
- Increased anxiety/stress
- Increased tiredness/fatigue
- Increased/decreased sleep

STEPS is a psychological intervention, not pharmacological, and so physical adverse reactions are not expected. Medical adverse events will be recorded using standard medical definitions.

21.4 Progression Criteria / Stopping Rules

The study will include an internal pilot study (the first 9-months of recruitment). There are no formal statistical stopping rules; progression rules for the internal pilot are described in Table 4 below. The trial may be prematurely discontinued by the Sponsor or Chief Investigator based on new safety information or for other reasons given by the DMC or PSC regulatory authority or ethics committee concerned.

Another reason for stopping the trial prematurely would be poor recruitment and engagement with the STEPS intervention or attrition during the pilot phase, which does not improve despite efforts to engage clinical teams and parents of referred children. This will be reviewed after the 9-month internal pilot. If the

internal pilot targets for recruitment, engagement or attrition are not met (below), the trial may be terminated on consensus advice of the PSC, DMC and the study funder.

The objective of the internal pilot is to determine whether recruitment, engagement with the intervention and retention to the trial are sufficient to allow the trial to progress and provide a definitive answer on the effectiveness of the intervention. Progression rules regarding recruitment, treatment engagement and attrition during the internal pilot are specified in Table 4. Briefly, green means that the trial will continue, amber – the research team will review ways of improving with the PSC and the Patient and Public Involvement (PPI) Steering Group. The decision to continue or stop the trial will be made independently by the PSC taking advice from the DMC.

Table 4. OPTIMA RCT internal pilot progression rules.

**RECRUITMENT**
**GREEN: > 70%** of recruitment target for that period.<br>**AMBER: 50-70%.** Lower rate would be also acceptable, if there's an evidence of an upward recruitment trajectory and/or a clear plan for further improvement (e.g., new sites, or ways of recruiting).<br>**RED: < 50%** and no evidence of an upward trajectory.
**INTERVENTION ENGAGEMENT**
**GREEN: > 90%** of participants providing primary outcome data at primary endpoint (3 months post-randomisation) will have engaged with the two first modules of the intervention.<br>**AMBER**: **60-90%.** Lower rate also acceptable if evidence of improvement or/and a clear plan for improving engagement.<br>**RED**: **< 60%** and no evidence of improvement.
**ATTRITION**
**GREEN: > 75%** of participants who have reached the relevant time window will have completed primary endpoint assessments to provide primary outcome data.<br>**AMBER: 50-75%.** Lower rate acceptable, if evidence of improving retention and/or clear plan for enhancing retention.<br>**RED: <50%** and no evidence of improving retention.

22. Auditing

Auditing is not applicable to this protocol.

23. Ethics & Regulatory Approvals

The trial will be conducted in compliance with the principles of the Declaration of Helsinki (1996), the principles of GCP and in accordance with all applicable regulatory requirements.

The research team and the sponsor will submit the study protocol, participant information sheet, consent form, and other relevant supporting documents to the North West - Liverpool Central Research Ethics Committee Research Ethics Committee (REC) and will ensure these have been approved by the appropriate regulatory bodies, prior to any participant recruitment. The protocol and all agreed substantial protocol amendments will be documented and submitted for ethical and regulatory approval prior to implementation.

The electronic version of this document is the latest version. It is responsibility of the individual to ensure that any paper material is the current version. Printed material is uncontrolled documentation.

No recruitment activity will take place before ethical approval for the study has been obtained. The CI and the sponsor will ensure that the main REC is notified that the study has finished within the appropriate timeframe. If the study is terminated prematurely, the REC will be notified within 15 days of the end of the study. The Chief Investigator will submit a final report at conclusion of the trial to the funder, the REC and the Sponsor.

24. Protocol amendments

In case of new information becoming available, which may result in significant changes to the risks and benefits of taking part, the Participant Information Sheet and informed consent form will be reviewed and updated accordingly. All participants actively enrolled in the study will be informed of the updated information and will be given a revised copy of the Participant Information Sheet and informed consent form to confirm their wish to continue taking part.

25. Consent

Participants in the study will be parents of children, who screened positive for high levels of hyperactivity/impulsivity, inattention, and conduct problems, children themselves (optional), and clinicians (qualitative interviews).

25.1 Parents

All participants will provide written informed consent to take part in the study. The informed consent form will be provided in electronic form using Red Pill ePRO system to be completed by the participant before they enter the study. A link to the electronic consent form will be emailed or texted to parents who wish to enrol in the study. The electronic consent form will include a link to the Parent Information Sheet. After answering consent questions, the participant will type in their name and provide a signature. The signature panel works with a mouse or touchscreen. On the consent form there will be a separate question asking parents to consent to a qualitative interview. Parents who do not consent to this will not be invited to take part in the interviews. Parents, who agree to take part in the interviews via the written consent form, will be asked to confirm their consent verbally before the interview takes place.

On submitting the signed consent form the participant is shown the form within Red Pill with their completed answers and signature. They are asked to check the box to make the declaration that they will electronically sign the consent on saving the form. On saving the form a time stamped PDF version of the consent form is created and stored in the system. It is also sent to the participant by email as a record of their consent. A copy of the completed form will be extracted by a research assistant and retained at the study site and placed in central file. A copy of the consent form will be shared with the child’s clinical team and a child’s GP will be informed of the study participation.

25.2 Children

Parents will provide consent on behalf of the children to take part in ESO (remote drawing task). Consent statements regarding a child’s participation in the study will be included in the electronic form completed by

parents via the Red Pill ePRO system. Parents will be emailed a Child Information Sheet when they are invited to participate, and they will be asked to share it with their child. Children's verbal assent will be obtained prior to completing the remote drawing task. We will not ask the children to provide written assent as the burden associated with the task is low. It is a drawing game completed with a parent in the comfort of a family home. Moreover, collecting children's assessment electronically will be challenging due to a young age of participants. Researchers will record child's verbal responses to assent questions on the assent form, sign the form and email to the parent for their record. Child's verbal assent will also be documented in the trial database.

25.3 Clinicians

All participants will provide written informed consent to take part in the study. The informed consent form will be provided in electronic form using Qualtrics. A link to the electronic consent form will be emailed to clinicians, who wish to take part in the interviews. The electronic consent form will include a link to the Clinician Information Sheet. After answering consent questions, the participant will confirm their consent by typing in their name in the response box. A copy of the completed consent form countersigned by the researcher will be emailed to the clinician and also retained at the study site and placed in the central study file.

25.4 Withdrawal of participants

We do not expect using STEPS to lead to harm that would suggest the intervention needs to be discontinued. However, parents will be able to withdraw from the trial at any point for any reason. Through the information sheet, parents will be informed that they can withdraw from the study either by informing the team that they wish to opt out of taking part in follow-ups or by ignoring the reminders to complete the follow-up surveys. They can also stop using STEPS and delete the app from their phone at any time.

If a participant actively informs the research team that they would like to withdraw from the study, it will initially be clarified which aspects of the study they wish to withdraw (i.e., intervention, data collection or data collection from records) from and they will then be sent a 'confirmation of withdrawal' email and will be entered in the Red Pill database as withdrawn from the study. Copy of the email confirming withdrawal will be retained in the trial master file. Information about withdrawal will be shared with the participant's local clinical team. Participants will be made aware in the participant information sheets that any non-identifiable data collected up to the point of their withdrawal may still be used in the analyses. After withdrawal has been confirmed, participants will not be requested to complete any further measures but will be asked by the research team to provide voluntary feedback regarding the reason for their withdrawal. If feedback is provided, this will be recorded in the database. Once a participant has withdrawn from the study it will not be possible to re-enter the study or resume using the app. Withdrawn participants will not be replaced into the study.

Participants will not be withdrawn from the study due to poor engagement with the app. If a participant does not continue using STEPS, we would still invite them to complete outcome measures unless they explicitly state they wish to withdraw. In this case participants will be informed it is still key to the success of the study to collect outcome measures, even if they do not want to use the app.

Failure to complete outcome measures at one follow-up time point will not imply withdrawal from the study, and the participants will still be invited to complete measures at the next follow-up time point, unless they explicitly state otherwise.

26. Confidentiality

In this study we will collect the following identifiable information: names, dates of birth, postal address, telephone number and email address. The STEPS app will collect administrative data (i.e., name, email address, mobile phone number), required to enable participants to access the STEPS app and to notify them about the updates.

The Chief Investigator and all members of the research team will take every effort to preserve the confidentiality of participants taking part in the study. To de-identify the data, each participant will be assigned a study ID. A reference file with the study IDs and participant identifiable information will be kept in a secure King’s College OneDrive for Business folder, which is a Microsoft 365 integrated cloud-based storage and will be backed up on an external encrypted hard drive.

Participants identifiable data required for administrative purposes (e.g., name and contact details) will be stored in a separate file from the data files. These will be accessed only by those members of the research team who are responsible for contacting participants (e.g., to email a link to the online survey). No individual participant’s data will be identifiable in the publications or reports that may result from this study. All personal data will be stored securely for seven years in line with the KCL record retention policy.

27. Publication Policy

A full and complete account of the trial results will be published in a high-quality peer reviewed scientific journal and full report for the funder. Authorship will be determined according to COPE standards based on the individual contributions. In addition, the findings will be disseminated through oral and poster presentations at a range of conferences and seminars in the UK and overseas. There will also be a general dissemination programme for clinicians, commissionaires and parents through the OPTIMA website.

28. Insurance / Indemnity

The lead sponsor, King's College London, will take primary responsibility for ensuring that the design of the study meets appropriate standards and that arrangements are in place to ensure appropriate conduct and reporting. King's College London also provides cover under its No Fault Compensation Insurance, which provides for payment of damages or compensation in respect of any claim made by a research subject for

bodily injury arising out of participation in a clinical trial or healthy volunteer study (with certain restrictions). The co-sponsor, South London and Maudsley NHS Foundation Trust, takes responsibility for ensuring that appropriate standards, conduct and reporting are adhered to regarding its facilities and staff involved with the project.

29. Financial Aspects

The study has been funded by the NIHR reference number RP-PG-0618-20003. The project funding covers all costs in relation to the OPTIMA programme, including but not limited to, higher education institutions costs, equipment and PPI cost. The funding covers the period from 1 March 2020 to 30 September 2025.

30. Signatures

___________________________________ ______________________

Chief Investigator Date

Print name

___________________________________ ______________________

Statistician (if applicable) Date

Print name

31. Appendices

APPENDIX 1

OPTIMA Assessment & Diagnosis Form

1. **Has the child been formally assessed by the services they were referred to?**

   Yes (go to question 2)
   No (end of form)

2. **Date of assessment:** dd/mm/yyyy

3. **Following their assessment, has the child received an ADHD diagnosis from the service they were initially referred to?**

   Yes (go to question 4)
   No (go to question 6)

4. **Date of diagnosis:** dd/mm/yyyy

5. **Has the child received another diagnosis?**

   Autism spectrum disorder (ASD)
   Conduct disorder
   Oppositional defiant disorder
   Other
   No diagnosis

6. **Has the child received treatment?**

   No treatment
   Yes, medication (Go to Q7)
   Yes, psychological treatment for the child
   Yes, parenting support

7. **Name of medication and dose** [free text 100 characters]

APPENDIX 2

Trial expectations questionnaire

In this questionnaire, we would like to find out about your general expectations and experience of parent training.

1. Have you ever taken part in a research study before (online or face to face)?
   Yes (1)

   No (0)

2. Have you ever taken part in a research study specifically on parenting before (online or face to face)?
   Yes (1)

   No (0)

3. Please rate your agreement with the following statements about parent training.
   Overall, I think parent training is/would be:

   Helpful to me as a parent
   Strongly disagree (1)
   Somewhat disagree (2)
   Not sure (3)
   Somewhat agree (4)
   Strongly agree (5)

   Helpful to my child
   Strongly disagree (1)
   Somewhat disagree (2)
   Not sure (3)
   Somewhat agree (4)
   Strongly agree (5)

   A good use of my time
   Strongly disagree (1)
   Somewhat disagree (2)
   Not sure (3)
   Somewhat agree (4)
   Strongly agree (5)

Not enough reward for the time it takes to get involved

Strongly disagree (1)

Somewhat disagree (2)

Not sure (3)

Somewhat agree (4)

Strongly agree (5)

A waste of time

Strongly disagree (1)

Somewhat disagree (2)

Not sure (3)

Somewhat agree (4)

Strongly agree (5)

4. We would like to find out about your *expectations* of using the STEPS parenting app. Based on what you have been told about it, please rate your agreement with the following statements about STEPS.

STEPS would help me feel better about my parenting

Strongly disagree (1)

Somewhat disagree (2)

Not sure (3)

Somewhat agree (4)

Strongly agree (5)

STEPS would help me manage my child better

Strongly disagree (1)

Somewhat disagree (2)

Not sure (3)

Somewhat agree (4)

Strongly agree (5)

STEPS would improve my knowledge about parenting

Strongly disagree (1)

Somewhat disagree (2)

Not sure (3)

Somewhat agree (4)

Strongly agree (5)

STEPS would improve my mental health
Strongly disagree (1)
Somewhat disagree (2)
Not sure (3)
Somewhat agree (4)
Strongly agree (5)

STEPS would improve our family life
Strongly disagree (1)
Somewhat disagree (2)
Not sure (3)
Somewhat agree (4)
Strongly agree (5)

STEPS would improve my child's behaviour
Strongly disagree (1)
Somewhat disagree (2)
Not sure (3)
Somewhat agree (4)
Strongly agree (5)

STEPS would improve my relationship with my child
Strongly disagree (1)
Somewhat disagree (2)
Not sure (3)
Somewhat agree (4)
Strongly agree (5)

STEPS would improve my child's mental health
Strongly disagree (1)
Somewhat disagree (2)
Not sure (3)
Somewhat agree (4)
Strongly agree (5)

STEPS would help my child feel happier
Strongly disagree (1)
Somewhat disagree (2)

Not sure (3)

Somewhat agree (4)

Strongly agree (5)

STEPS would help my child do better at school

Strongly disagree (1)

Somewhat disagree (2)

Not sure (3)

Somewhat agree (4)

Strongly agree (5)

5. Please use the text box below to tell us more about your hopes and/or worries about using the STEPS parenting app.
   [free text 500 characters]

6. If you could choose, would you prefer face to face parent training (where you meet in person with a therapist who explains things to you) or online parent training delivered via an app (such as STEPS)?

   Face to face (1)

   Online (2)

   No preference, as long as I get help and support (3)

APPENDIX 3

Medical and psychological events and difficulties questionnaire

We want to keep track of whether there are any potential concerns about taking part in OPTIMA. These questions will ask about any **physical** and **mental health difficulties your child** and **you** may have experienced in the last **3 months**. We will also ask about any difficulties **your child** and **you** may have had with daily activities.

This section will ask about any **physical health** or **medical** problems. We will first ask about **your child** and then ask about **you**.

1. Has **your child** had any **physical health** or **medical** problems in the last 3 months?

   NO: go to Q2
   YES:
   - Please say a bit more about the problem [500 characters]
   - When did this problem start? Please provide approximate date.
   - When did this problem end? Please provide approximate day or select 'ongoing' if not resolved.
   - Did this problem result in hospitalisation? **Yes/No.** If yes, please describe [500 characters].

2. Have **you** had any **physical health** or **medical** problems in the last 3 months?

   NO: go to Q3
   YES
   - Please say a bit more about the problem [500 characters]
   - When did this problem start? Please provide approximate date.
   - When did this problem end? Please provide approximate day or select 'ongoing' if not resolved.
   - Did this problem result in hospitalisation? Yes/No. If yes, please describe [500 characters].

This section will ask about any **mental health** or **behavioural** problems. We will first ask about **your child** and then ask about **you**.

3. Has your child had any **mental health** or **behavioural** problems in the last 3 months?

   NO: go to Q4
   YES
   - Please say a bit more about the problem [500 characters]
   - When did this problem start? Please provide approximate date.
   - When did this problem end? Please provide approximate day or select 'ongoing' if not resolved.
   - Did this problem result in hospitalisation? **Yes/No.** If yes, please describe [500 characters].

4. Have you had any **mental health** problems in the last 3 months?

   NO: go to Q5
   YES:
   - Please say a bit more about the problem [500 characters]
   - When did this problem start? Please provide approximate date.
   - When did this problem end? Please provide approximate day or select 'ongoing' if not resolved.
   - Did this problem result in hospitalisation? **Yes/No.** If yes, please describe [500 characters].

This section will ask about any **difficulties in relationships with family members you live with** or **daily activities**. We will first ask about **your child** and then ask about **you**.

5. Has your child had any **difficulties in relationships with family members you live with** or **daily activities** at home, school, including online schooling or things that they normally do in the last 3 months?
   NO: go to Q4
   YES

- Please say a bit more about the problem [500 characters]
- When did this problem start? Please provide approximate date.
- When did this problem end? Please provide approximate day or select 'ongoing' if not resolved.

6. Have you had any **difficulties in relationships with family members you live with** or **daily activities** at home, work or other things that you normally do in the last 3 months?

   NO: go to Q7
   YES

- Please say a bit more about the problem [500 characters]
- When did this problem start? Please provide approximate date.
- When did this problem end? Please provide approximate day or select 'ongoing' if not resolved.

7. Have any of the difficulties you described given you concerns about taking part in OPTIMA?

   NO: go to Q8
   YES: please describe [500 characters]

8. Did anything good happen to you or your child because of taking part in OPTIMA? (Any specific good things that have happened or things that have changed in a positive way to do with or because of taking part).

   NO: end of form
   YES: please describe [500 characters]

APPENDIX 4

Adverse Events Form

Adverse Events Form

This form was completed based on:

1. A report from a research assistant?
2. A completion of an online questionnaire?

**1. Details of adverse event (describe below what has been reported)**<br>[free text up to 500 characters]	**2. Medical/physical health**<br>1. Cardiovascular<br>2. Respiratory<br>3. Hepatic<br>4. Gastro-intestinal<br>5. Genito-urinary/renal<br>6. Endocrine<br>7. Haematological<br>8. Musculo-skeletal<br>9. Neoplasia<br>10. Neurological<br>11. Psychological<br>12. Immunological<br>13. Dermatological<br>14. Allergies<br>15. Eyes, ear, nose, throat	**3. Mental health/ behaviour**<br>**0.** No<br>**1.** Yes	**4. Relationships or daily activities difficulties**<br>**0.** No<br>**1.** Yes	**5. Start date**	**6. Outcome**<br>**1.** Resolved<br>**2.** Resolved with treatment/ intervention<br>**3.** Ongoing	**7. End date**	**8. Relationship to intervention**<br>**1.** Definitely related<br>**2.** Likely<br>**3.** Possibly<br>**4.** Unlikely<br>**5.** Not related	**9. Expected?**<br>**1.** Expected<br>**2.** Unexpected	**10. Is this event serious?**<br>**0.** No<br>**1.** Results in death<br>**2.** Is life-threatening<br>**3.** Requires hospitalisation or prolongation of existing hospitalisation<br>**4.** Persistent or significant disability or incapacity<br>**5.** Consists of a congenital anomaly or birth defect	**11. SUSAR?**<br>**0.** No<br>**1.** Yes	**12. Who is involved?**<br>**1.** Target child<br>**2.** Target parent	**13. Has the event led to the family no longer participating in the study?**<br>**0.** No<br>**1.** Yes

References

Angrist, J. D., Imbens, G. W., & Rubin, D. B. (1996). Identification of causal effects using instrumental variables. *Journal of the American statistical Association, 91*(434), 444-455.

Arnold, D. S., O'Leary, S. G., Wolff, L. S., & Acker, M. M. (1993). The Parenting Scale: a measure of dysfunctional parenting in discipline situations. *Psychological assessment, 5*(2), 137.

Assmann, S. F., Pocock, S. J., Enos, L. E., & Kasten, L. E. (2000). Subgroup analysis and other (mis) uses of baseline data in clinical trials. *The lancet, 355*(9209), 1064-1069.

Barrett, B., Byford, S., Sharac, J., Hudry, K., Leadbitter, K., Temple, K., . . . consortium, P. (2012). Service and wider societal costs of very young children with autism in the UK. *Journal of autism and developmental disorders, 42*(5), 797-804.

Berument, S. K., Rutter, M., Lord, C., Pickles, A., & Bailey, A. (1999). Autism screening questionnaire: diagnostic validity. *The British journal of psychiatry, 175*(5), 444-451.

Bourke-Taylor, H. M., Pallant, J. F., & Law, M. (2014). Update on the Child's Challenging Behaviour Scale following evaluation using Rasch analysis. *Child: Care, Health and Development, 40*(2), 242-249. doi:https://doi.org/10.1111/cch.12035

Brannan, A. M., Heflinger, C. A., & Bickman, L. (1997). The Caregiver Strain Questionnaire:Measuring the Impact on the Family of Living with a Child with Serious Emotional Disturbance. *Journal of Emotional and Behavioral Disorders, 5*(4), 212-222. doi:10.1177/106342669700500404

Braun, V., & Clarke, V. (2013). *Successful qualitative research: A practical guide for beginners*: sage.

Breider, S., de Bildt, A., Nauta, M. H., Hoekstra, P. J., & van den Hoofdakker, B. J. (2019). Self-directed or therapist-led parent training for children with attention deficit hyperactivity disorder? A randomized controlled non-inferiority pilot trial. *Internet interventions, 18*, 100262.

Briggs, A. H., Wonderling, D. E., & Mooney, C. Z. (1997). Pulling cost-effectiveness analysis up by its bootstraps: a non-parametric approach to confidence interval estimation. *Health economics, 6*(4), 327-340.

Bussing, R., Fernandez, M., Harwood, M., Hou, W., Garvan, C. W., Eyberg, S. M., & Swanson, J. M. (2008). Parent and teacher SNAP-IV ratings of attention deficit hyperactivity disorder symptoms: Psychometric

properties and normative ratings from a school district sample. *Assessment*.

Canaway, A. G., & Frew, E. J. (2013). Measuring preference-based quality of life in children aged 6–7 years: a comparison of the performance of the CHU-9D and EQ-5D-Y—the WAVES Pilot Study. *Quality of life research, 22*(1), 173-183.

Commissioner, C. s. (2016). Lightning review: access to child and adolescent mental health services.

Committee, H. o. C. H. (2014). Children's and adolescents' mental health and CAMHS. *Third report of Session, 15*.

Daley, D., Van der Oord, S., Ferrin, M., Danckaerts, M., Doepfner, M., Cortese, S., & Sonuga-Barke, E. J. (2015). The impact of behavioral interventions for children and adolescents with attention deficit hyperactivity disorder: a meta-analysis of randomized controlled trials across multiple outcome domains. *Journal of the American Academy of Child and Adolescent Psychiatry*.

Dalrymple, R. A., Maxwell, L. M., Russell, S., & Duthie, J. (2019). NICE guideline review: Attention deficit hyperactivity disorder: diagnosis and management (NG87). *Archives of Disease in Childhood-Education and Practice*.

Dalsgaard, S., Mortensen, P. B., Frydenberg, M., & Thomsen, P. H. (2013). Long-term criminal outcome of children with attention deficit hyperactivity disorder. *Criminal Behaviour and Mental Health, 23*(2), 86-98.

Driscoll, K., & Pianta, R. C. (2011). Mothers' and Fathers' Perceptions of Conflict and Closeness in Parent-Child Relationships during Early Childhood. *Journal of Early Childhood & Infant Psychology*(7).

Dunn, G., Emsley, R., Liu, H., Landau, S., Green, J., White, I., & Pickles, A. (2015). Evaluation and validation of social and psychological markers in randomised trials of complex interventions in mental health: a methodological research programme. *Health Technology Assessment (Winchester, England), 19*(93), 1-115, v.

DuPaul, G. J., Kern, L., Belk, G., Custer, B., Daffner, M., Hatfield, A., & Peek, D. (2018). Face-to-face versus online behavioral parent training for young children at risk for ADHD: treatment engagement and outcomes. *Journal of Clinical Child & Adolescent Psychology, 47*(sup1), S369-S383.

Erskine, H. E., Ferrari, A. J., Nelson, P., Polanczyk, G. V., Flaxman, A. D., Vos, T., . . . Scott, J. G. (2013). Research Review: Epidemiological modelling of attention-deficit/hyperactivity disorder and conduct disorder for the Global Burden of Disease Study 2010. *Journal of Child Psychology and Psychiatry, 54*(12), 1263-1274.

The electronic version of this document is the latest version. It is responsibility of the individual to ensure that any paper material is the current version. Printed material is uncontrolled documentation.

Fenwick, E., & Byford, S. (2005). A guide to cost-effectiveness acceptability curves. *The British journal of psychiatry, 187*(2), 106-108.

Fletcher, J. M. (2014). The effects of childhood ADHD on adult labor market outcomes. *Health economics, 23*(2), 159-181.

Franke, N., Keown, L. J., & Sanders, M. R. (2020). An RCT of an online parenting program for parents of preschool-aged children with ADHD symptoms. *Journal of attention disorders, 24*(12), 1716-1726.

Groenman, A. P., Hornstra, R., Hoekstra, P. J., Steenhuis, L., Aghebati, A., Boyer, B. E., . . . van den Hoofdakker, B. J. (2021). An Individual Participant Data Meta-analysis: Behavioral Treatments for Children and Adolescents With Attention-Deficit/Hyperactivity Disorder. *J Am Acad Child Adolesc Psychiatry*. doi:10.1016/j.jaac.2021.02.024

Hall, C. L., Guo, B., Valentine, A. Z., Groom, M. J., Daley, D., Sayal, K., & Hollis, C. (2020). The validity of the SNAP-IV in children displaying ADHD symptoms. *Assessment, 27*(6), 1258-1271.

Herdman, M., Gudex, C., Lloyd, A., Janssen, M., Kind, P., Parkin, D., . . . Badia, X. (2011). Development and preliminary testing of the new five-level version of EQ-5D (EQ-5D-5L). *Quality of life research, 20*(10), 1727-1736.

Johnston, C. (1996). Parent characteristics and parent-child interactions in families of nonproblem children and ADHD children with higher and lower levels of oppositional-defiant behavior. *Journal of abnormal child psychology, 24*(1), 85-104.

Johnston, C., & Mash, E. J. (1989). A measure of parenting satisfaction and efficacy. *Journal of clinical child psychology, 18*(2), 167-175.

Lundahl, B., Risser, H. J., & Lovejoy, M. C. (2006). A meta-analysis of parent training: Moderators and follow-up effects. *Clinical Psychology Review, 26*(1), 86-104. doi:https://doi.org/10.1016/j.cpr.2005.07.004

McGarry, J., McNicholas, F., Buckley, H., Kelly, B. D., Atkin, L., & Ross, N. (2008). The clinical effectiveness of a brief consultation and advisory approach compared to treatment as usual in child and adolescent mental health services. *Clinical child psychology and psychiatry, 13*(3), 365-376.

Mind, Y. I. Scoring the Strengths and Difficulties Questionnaire for ages 4–17. Retrieved from https://www.sdqinfo.org/py/sdqinfo/c0.py

Moore, G. F., Audrey, S., Barker, M., Bond, L., Bonell, C., Hardeman, W., . . . Wight, D. (2015). Process evaluation of complex interventions: Medical Research Council guidance. *bmj, 350*.

Moyá, J., Stringaris, A. K., Asherson, P., Sandberg, S., & Taylor, E. (2014). The impact of persisting hyperactivity on social relationships: A

The electronic version of this document is the latest version. It is responsibility of the individual to ensure that any paper material is the current version. Printed material is uncontrolled documentation.

community-based, controlled 20-year follow-up study. *Journal of attention disorders, 18*(1), 52-60.

Ohan, J. L., Leung, D. W., & Johnston, C. (2000). The Parenting Sense of Competence scale: Evidence of a stable factor structure and validity. *Canadian Journal of Behavioural Science/Revue canadienne des sciences du comportement, 32*(4), 251.

Oliver, B. R., & Pike, A. (2021). Introducing a Novel Online Observation of Parenting Behavior: Reliability and Validation. *Parenting, 21*(2), 168-183. doi:10.1080/15295192.2019.1694838

Pianta, R. C. (1992). Child-parent relationship scale. *Unpublished measure, University of Virginia, 11*, 39-41.

Podolski, C.-L., & Nigg, J. T. (2001). Parent stress and coping in relation to child ADHD severity and associated child disruptive behavior problems. *Journal of clinical child psychology, 30*(4), 503-513.

Polderman, T., Boomsma, D., Bartels, M., Verhulst, F., & Huizink, A. (2010). A systematic review of prospective studies on attention problems and academic achievement. *Acta Psychiatrica Scandinavica, 122*(4), 271-284.

Rhoades, K. A., & O'Leary, S. G. (2007). Factor structure and validity of the parenting scale. *Journal of Clinical Child and Adolescent Psychology, 36*(2), 137-146.

Sayal, K., Taylor, E., & Beecham, J. (2003). Parental perception of problems and mental health service use for hyperactivity. *Journal of the American Academy of Child & Adolescent Psychiatry, 42*(12), 1410-1414.

Sonuga-Barke, E. J., Barton, J., Daley, D., Hutchings, J., Maishman, T., Raftery, J., . . . Coghill, D. (2018). A comparison of the clinical effectiveness and cost of specialised individually delivered parent training for preschool attention-deficit/hyperactivity disorder and a generic, group-based programme: a multi-centre, randomised controlled trial of the New Forest Parenting Programme versus Incredible Years. *European child & adolescent psychiatry, 27*(6), 797-809.

Sonuga-Barke, E. J., Daley, D., Thompson, M., Laver-Bradbury, C., & Weeks, A. (2001). Parent-based therapies for preschool attention-deficit/hyperactivity disorder: a randomized, controlled trial with a community sample. *Journal of the American Academy of Child & Adolescent Psychiatry, 40*(4), 402-408.

Swanson, J. M., Kraemer, H. C., Hinshaw, S. P., Arnold, L. E., Conners, C. K., Abikoff, H. B., . . . Greenhill, L. L. (2001). Clinical relevance of the primary findings of the MTA: Success rates based on severity of ADHD and ODD symptoms at the end of treatment. *Journal of the American Academy of Child & Adolescent Psychiatry*.

The electronic version of this document is the latest version. It is responsibility of the individual to ensure that any paper material is the current version. Printed material is uncontrolled documentation.

Telford, C., Green, C., Logan, S., Langley, K., Thapar, A., & Ford, T. (2013). Estimating the costs of ongoing care for adolescents with attention-deficit hyperactivity disorder. *Social psychiatry and psychiatric epidemiology, 48*(2), 337-344.

Theule, J., Wiener, J., Rogers, M. A., & Marton, I. (2011). Predicting parenting stress in families of children with ADHD: Parent and contextual factors. *Journal of Child and Family studies, 20*(5), 640-647.

Weitlauf, A. S., Vehorn, A. C., Taylor, J. L., & Warren, Z. E. (2014). Relationship satisfaction, parenting stress, and depression in mothers of children with autism. *Autism, 18*(2), 194-198.

Zou, G. (2004). A Modified Poisson Regression Approach to Prospective Studies with Binary Data. *American Journal of Epidemiology, 159*(7), 702-706. doi:10.1093/aje/kwh090

The electronic version of this document is the latest version. It is responsibility of the individual to ensure that any paper material is the current version. Printed material is uncontrolled documentation.
